# A Multifactorial Weight Reduction Programme for Children with Overweight and Asthma: A Randomized Controlled Trial

**DOI:** 10.1371/journal.pone.0157158

**Published:** 2016-06-13

**Authors:** Maartje Willeboordse, Kim D. G. van de Kant, Frans E. S. Tan, Sandra Mulkens, Julia Schellings, Yvonne Crijns, Liesbeth van der Ploeg, Constant P. van Schayck, Edward Dompeling

**Affiliations:** 1 Department of Paediatric Pulmonology, School for Public Health and Primary Care (CAPHRI), Maastricht University Medical Centre (MUMC), Maastricht, the Netherlands; 2 Department of Methodology and Statistics, CAPHRI, MUMC, Maastricht, the Netherlands; 3 Department of Clinical Psychological Science, School for Experimental Psychopathology (EPP), Maastricht University, Faculty of Psychology and Neuroscience, Maastricht, the Netherlands; 4 Department of Physiotherapy, MUMC, Maastricht, the Netherlands; 5 Department of Dietetics, MUMC, Maastricht, the Netherlands; 6 Department of Family Medicine, CAPHRI, MUMC, Maastricht, the Netherlands; Vanderbilt University, UNITED STATES

## Abstract

**Background:**

There is increasing evidence that obesity is related to asthma development and severity. However, it is largely unknown whether weight reduction can influence asthma management, especially in children.

**Objective:**

To determine the effects of a multifactorial weight reduction intervention on asthma management in overweight/obese children with (a high risk of developing) asthma.

**Methods:**

An 18-month weight-reduction randomized controlled trial was conducted in 87 children with overweight/obesity and asthma. Every six months, measurements of anthropometry, lung function, lifestyle parameters and inflammatory markers were assessed. Analyses were performed with linear mixed models for longitudinal analyses.

**Results:**

After 18 months, the body mass index-standard deviation score decreased by -0.14±0.29 points (p<0.01) in the intervention group and -0.12±0.34 points (p<0.01) in the control group. This change over time did not differ between groups (p>0.05). Asthma features (including asthma control and asthma-related quality of life) and lung function indices (static and dynamic) improved significantly over time in both groups. The FVC% predicted improved over time by 10.1 ± 8.7% in the intervention group (p<0.001), which was significantly greater than the 6.1 ± 8.4% in the control group (p<0.05).

**Conclusions & clinical relevance:**

Clinically relevant improvements in body weight, lung function and asthma features were found in both the intervention and control group, although some effects were more pronounced in the intervention group (FVC, asthma control, and quality of life). This implies that a weight reduction intervention could be clinically beneficial for children with asthma.

**Trial Registration:**

ClinicalTrials.gov NCT00998413

## Introduction

The high childhood obesity prevalence is a major public health problem [1]. A high body mass index (BMI) increases the risk of developing asthma. Prospective studies suggested that a high BMI precedes asthma symptoms [2]. Moreover, a dose-response effect of BMI on asthma symptoms has been detected [3]. In comparison to their lean counterparts, the obese-asthmatic population is characterised by decreased asthma control, high medication use, and frequent asthma-related hospital admissions [4–6]. As obese children often become obese adults, it is necessary to tackle obesity early in life [7].

It is largely unknown whether weight reduction in overweight/obese children can improve asthma features (symptoms, lung function, amount of medication) in patients with already established disease, or can prevent the development of asthma in children at risk (e.g. with the combination of obesity and a first degree family member with asthma) [8, 9]. Most weight reduction studies in asthmatic patients have been conducted in adults [8, 9]. After weight reduction, improvements in lung function, asthma symptoms, asthma control and use of rescue medication have been found. However, most studies have sample sizes < 40 participants and weight loss was frequently achieved with bariatric surgery [8]. In children, a handful of studies have been performed [10–13]. These studies provided some interesting evidence that weight loss can induce beneficial changes in childhood asthma outcomes, such as asthma-related quality of life (QOL), asthma control, and improvements in lung function [10–13]. Although these first results are promising, the majority of these paediatric studies had small sample sizes (e.g. < 40 subjects) and/or no control group. Therefore, a well-powered randomized controlled trial (RCT) with a long term follow-up is needed to validate these findings.

In addition to methodological issues, the mechanisms underlying the increased risk of asthma in overweight/obese children, and/or the improvement in asthma features following weight loss, are poorly understood. Obesity causes mechanical, metabolic, and immunological changes that may affect the respiratory system [9]. Both mechanical changes (including improvements in chest wall compliance as a result of less mass loading of fat in and around the chest wall) and inflammatory changes (including increases in adiponectin, reductions in c-reactive protein and leptin) have been proposed to bring about improvement in asthma after weight reduction [9, 10, 14, 15]. Nevertheless, limited weight reduction studies in asthmatic children investigated the outcome parameters of both pathways [8, 10].

The primary aim of this study was to investigate the effects of a longitudinal, multidisciplinary, RCT directed towards weight reduction in overweight/obese children with (a high risk of developing) asthma. Our primary outcomes included the forced expiratory volume in 1 second (FEV_1_% predicted) and the BMI-standard deviation score (SDS). Secondary outcomes were other asthma features (asthma control, medication, asthma related QOL), lifestyle parameters (e.g. aerobic fitness), lung function indices, and inflammatory mediators (serum leptin and adiponectin).

## Methods

### Study design

**Fig 1:**
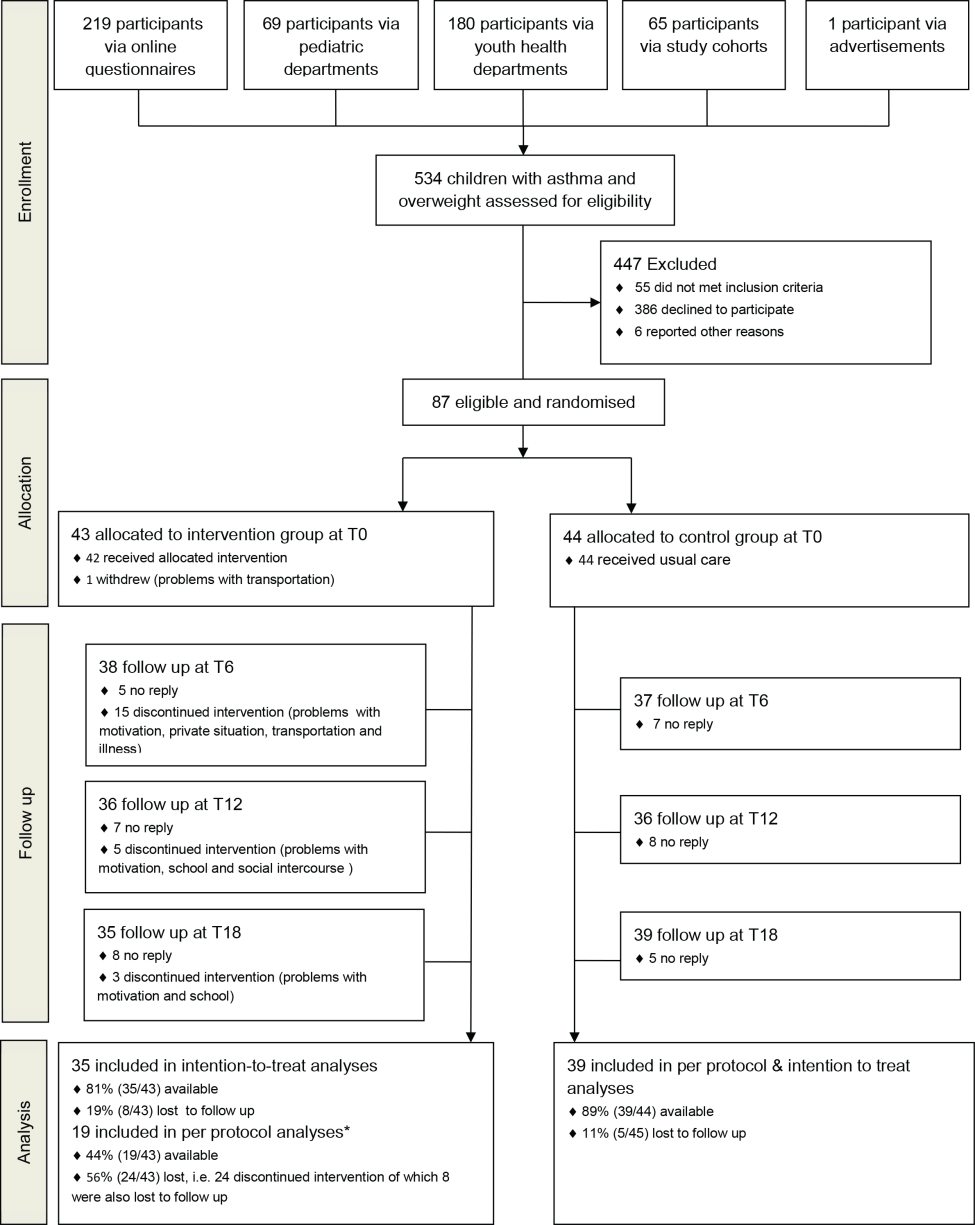
Consort flow diagram. * Two participants discontinued the sport sessions during the follow-up phase of the intervention due to membership of an organised sports association, but did continue to attend the lifestyle, individual and parental sessions. Those participants were included in the per protocol analysis.

Children were included in a multifactorial RCT entitled MIKADO (clinicaltrial.gov NCT00998413) [16]. Generalised block randomization with 10 participants per block, was performed by a computer program, with an allocation ratio of 1:1 [16]. Because this was a lifestyle study, blinding was not possible. The study duration was 18 months and regular measurements were performed every six months (T0, T6, T12, T18) (Fig 1). The control group received the usual care according to the international GINA (Global Initiative For Asthma) criteria and the Dutch national standard [17, 18]. All caregivers and children aged 12 years or older provided written informed consent prior to inclusion. The study was approved by the Medical Ethics Committee of Maastricht University Medical Centre (MEC 09-2-088).

### Study sample

Overweight/obese children (according to Dutch reference charts [19]) aged 6 to 16 were selected with either a clear clinical diagnosis of asthma, or with a high risk of developing asthma (because of the combination of overweight/obesity and at least one first-grade family member with asthma). Children with asthma fulfilled the GINA criteria for a diagnosis of asthma, with presence of asthma symptoms, bronchial hyperresponsiveness, and airway reversibility to a beta-2 agonist [17, 18] (Fig 1).

### The intervention

The control group received the usual care as stated above. In addition to the usual care, the intervention group received an 18-month multifactorial weight intervention consisting of 18 lifestyle sessions (including dietary advices and cognitive behavioural therapy), 10 parental sessions, 8 individual sessions, and regular sport sessions, at the MUMC+. The intervention was divided into an initial phase (months 0–6) and a follow-up phase (months 7–18). During the first six months the main themes of the lifestyle, parental and individual sessions were awareness of the obesity problem, consideration of new healthy behaviours and decision-making regarding new healthy behaviours. In the last 12 months of the programme children learned how to preserve their new behaviours and how to avoid reverting to old behaviours. The intervention was based on the ‘Health counselling model’. Sport sessions (60 minutes per session) consisted of regular group exercises (twice a week during the initial phase, and three times a month plus encouraging participation in an individual sport activity once a week, during the follow-up phase). A detailed description of the intervention is provided in a previous publication of the study protocol (S1 File. Study protocol) [16].

### Measurements

All outcome measures have been described previously in detail [16]. A summary is given below.

#### Primary outcome variables

The FEV_1_% predicted was obtained via maximal expiratory flow volume curves, measured with a spirometer (ZAN Messgerate, Oberthulba, Germany) according to the American Thoracic Society/European Respiratory Society (ATS/ERS) guidelines with predictive values of Zapletal et al., as described in detail previously [16, 20, 21]. Weight and height were measured twice and averaged to calculate the BMI (weight/height^2^). BMI-SDS was based on the reference charts of the Dutch fourth Nationwide Growth Study [22]. Overweight was defined as a BMI-SDS of ≥1.1 and <2.3, and obesity as a BMI-SDS of ≥2.3 [23].

#### Secondary outcome variables

Secondary outcome variables included asthma control [24, 25], asthma-related QOL [26], exercise-induced-bronchoconstriction (EIB), forced vital capacity (FVC% predicted), static lung function indices [20], and leptin and adiponectin serum concentration. Asthma was defined as uncontrolled if participants had a childhood Asthma Control Test (c-ACT) score of ≤19 [27]. EIB was measured as the difference in FEV_1_ before and 10 and 30 minutes after a maximal ergometer test. Static lung function indices were determined by body plethysmography (Viasys, Hoechberg, Germany) according to the ATS/ERS guidelines [20]. Non-fasting serum concentrations of leptin and adiponectin were determined by using a commercially available enzyme-linked immunosorbent assay (ELISA) (DY398 Duoset, R&D Systems, Minneapolis, USA) and radioimmunoassay (RIA) (HADP-61HK, Millicore corporation, Billerica, USA). All samples underwent duplicate testing.

#### Baseline and lifestyle

Other relevant outcome variables included comorbidities of asthma and obesity, step count, aerobic capacity (VO_2_peak% predicted) [28], dietary intake and atopic status.

Step count was defined as the average step count per day measured over the course of seven days by a tri-axial accelerometer (Yamax EX510 Power Walker, Yamax, Tokyo, Japan). A maximal incremental ergometer test was used to determine maximum aerobic capacity (VO_2_peak) with predicted values of Bongers et al [28]. Dietary intake was measured by means of three-day food records. Dietary intake was ranked on quality (on a scale from zero to 16) based on amount of principal meals, fruit consumption, unhealthy snacks and healthy milk consumption. The Dutch guidelines for healthy paediatric dietary intake were used as a template for this ranking scale which was developed by our multidisciplinary team [29]. Atopy was defined as an IgE serum concentration of ≥0.35 kU/L for a specific mixture for inhalant allergens (Phadiatop test, Pharmacia, Uppsala, Sweden).

### Statistical analysis

All data were assessed for normality. The power analysis was described previously [16]. Results are presented as mean ± SD unless stated otherwise. Loss to follow up was defined as discontinuation of the measurements, regardless of completion of the intervention (Fig 1). Non-compliance was defined as discontinuation of the intervention program, regardless of completion of the measurements. Participants who were lost to follow up were considered missing at random, as loss to follow up status (yes/no) was not associated with the most important baseline characteristics, and change in BMI-SDS and FEV_1_% predicted.

For all analyses, linear mixed model analysis techniques were used, as this technique corrects for the correlation with individuals in groups, which occurs in repeated-measures RCT designs. Another advantage of this technique is that it obviously accounts for missing values, in case data are missing at random. For the primary analysis, two linear mixed models were developed with both FEV_1_% predicted and BMI-SDS as dependent factors. Both intention to treat (with all participants who completed the measurements) and per-protocol analyses (with only participants who continued the intervention) were performed. Results are shown for intention to treat analyses, unless stated otherwise. An extensive description of all statistical models is provided in the supporting material (S2 File. Statistical analyses). A CONSORT statement is provided in the supporting material (S3 File. CONSORT statement).

## Results

### Recruitment and participant flow

Eighty-seven children were eligible and were randomized into the control group (n = 44) or intervention group (n = 43) (Fig 1). At 18 months, 39 patients were still followed up in the control group, and 35 patients in the intervention group. The study was performed between September 2011 and October 2013.

The presence at sport, group, parental and individual intervention sessions was good, averaging 71%, 65%, 63% and 68%, respectively. The average heart rate for each sport session was 149 ± 9 beats per minute, which correspondents to 79% of maximum heart rate. In total, 24 participants (56%) did not complete the intervention (Fig 1). The majority of the discontinuers (63%) stopped during the initial phase of the intervention.

In the control group, 29 participants reported that they received professional help to facilitate weight reduction during the 18-month study period (e.g. help of a dietician, a weight reduction consultant, specialised obesity care in a hospital, other weight reduction programmes, or online weight reduction advice).

### Baseline characteristics

Baseline characteristics are shown in Table 1. No relevant differences between intervention and control group were observed. In total, 78% of the children had asthma, half of the children were classified as obese, and 43% of the population used inhaled corticosteroids (ICS). Additional baseline characteristics are provided in the supporting material (S1 Table. Additional baseline characteristics).

**Table 1:** Baseline characteristics.

	Total group (n = 87)	Intervention group (n = 43)	Control group (n = 44)
*Baseline characteristics*			
Age in years, median (IQR)	12.2 (3.5)	12.3 (14.5)	11.9 (2.5)
Males, n/N* (% male)	53/87 (61)	26/43 (61)	27/44 (61)
Parents with a low education level, n/N* (%)	30/87 (34)	21/43 (49)	9/44 (20)
Average parental BMI, median (IQR)	27.7 (6.6)	26.9 (6.8)	28.4 (6.2)
*Anthropometrics*			
BMI in kg/m^2^, median (IQR)	25.2 (5.3)	25.0 (6.6)	25.2 (5.1)
BMI-SDS, mean (SD)	2.34 (0.55)	2.37 (0.58)	2.32 (0.52)
Obese children (BMI-SDS≥2.3), n/N* (%)	45/87 (52)	23/43 (53)	22/44 (50)
Skin fold thickness in cm, median (IQR)	129.8 (59.0)	125.0 (75.5)	130.0 (45.5)
Waist circumference in cm, median (IQR)	92.9 (16.1)	90.5 (19.5)	94.0 (10.4)
*Asthma features*			
Diagnosed asthma, n/N* (%)	68/87 (78)	32/43 (74)	36/44 (82)
Atopic, n/N* (%)	16/67 (24)	9/34 (27)	7/33 (21)
FEV_1_ in % predicted, mean (SD)	90.0 (15.2)	90.4 (13.6)	89.7 (16.8)
FVC in % predicted, mean (SD)	93.9 (11.4)	93.8 (10.1)	94.0 (12.7)
FEV_1_ /FVC in %, mean (SD)	80.3 (8.5)	80.6 (7.6)	80.1 (9.4)
ERV in % predicted, mean (SD)	79.2 (25.3)	75.9 (26.2)	82.1 (24.5)
PC_20_ histamine, median (IQR)	1.81 (4.92)	2.08 (4.31)	1.40 (5.57)
Uncontrolled asthma according to (c)-ACT, n/N* (%)†	23/68 (34)	11/32 (34)	12/36 (33)
PAQLQ, median (IQR) †	6.26 (0.87)	6.17 (0.72)	6.30 (1.35)
Use of SABA, n/N* (%)†	29/68 (43)	14/32 (44)	15/36 (42)
Use of more than one dose equivalent SABA, n/N* (%)†, ƍ	16/68 (24)	11/32 (34)	5/36 (14)
Use of ICS, n/N* (%)†	29/68 (43)	10/32 (31)	19/36 (53)
Use of more than one dose equivalent ICS, n/N* (%)†,ƍ	12/68 (18)	3/32 (9)	9/36 (25)
*Inflammatory parameters*			
FeNO in ppb, median (IQR)	15.8 (30.7)	15.0 (34.0)	16.8 (29.5)
Serum leptin in ng/ml, median (IQR)	1.83 (2.45)	1.83 (2.40)	1.83 (2.49)
Serum adiponectin in mg/mL, mean (SD)	11.6 (4.2)	11.2 (4.6)	12.0 (3.7)

* n/N: Number of participants with a positive outcome for this parameter/Number of participants measured. Numbers (N) may not add up to 87, 43 and 44, respectively due to missing values or, in case of asthma related parameters (†), as these parameters were only demonstrated for asthmatic participants (not for participants with a high risk of developing asthma).

ƍ Dose equivalents were calculated according to standard dosages of SABA and ICS, as described previously [16].

Abbreviations: (c)-ACT: (childhood) asthma control test, BMI: body mass index, BMI-SDS: body mass index standard deviation score, ERV: expiratory reserve volume, FeNO: fraction of exhaled nitric oxide, FEV_1_: forced expiratory volume in 1 second, FVC: forced vital capacity, ICS: inhaled corticosteroids, IQR: inter quartile range, PAQLQ: paediatric asthma quality of life questionnaire, PC_20_ histamine: histamine concentration that led to a drop of 20% in FEV_1,_ SABA: short-acting beta-2 agonists, SD: standard deviation.

### Primary research question: FEV_1_ and BMI-SDS (Fig 2)

**Fig 2:**
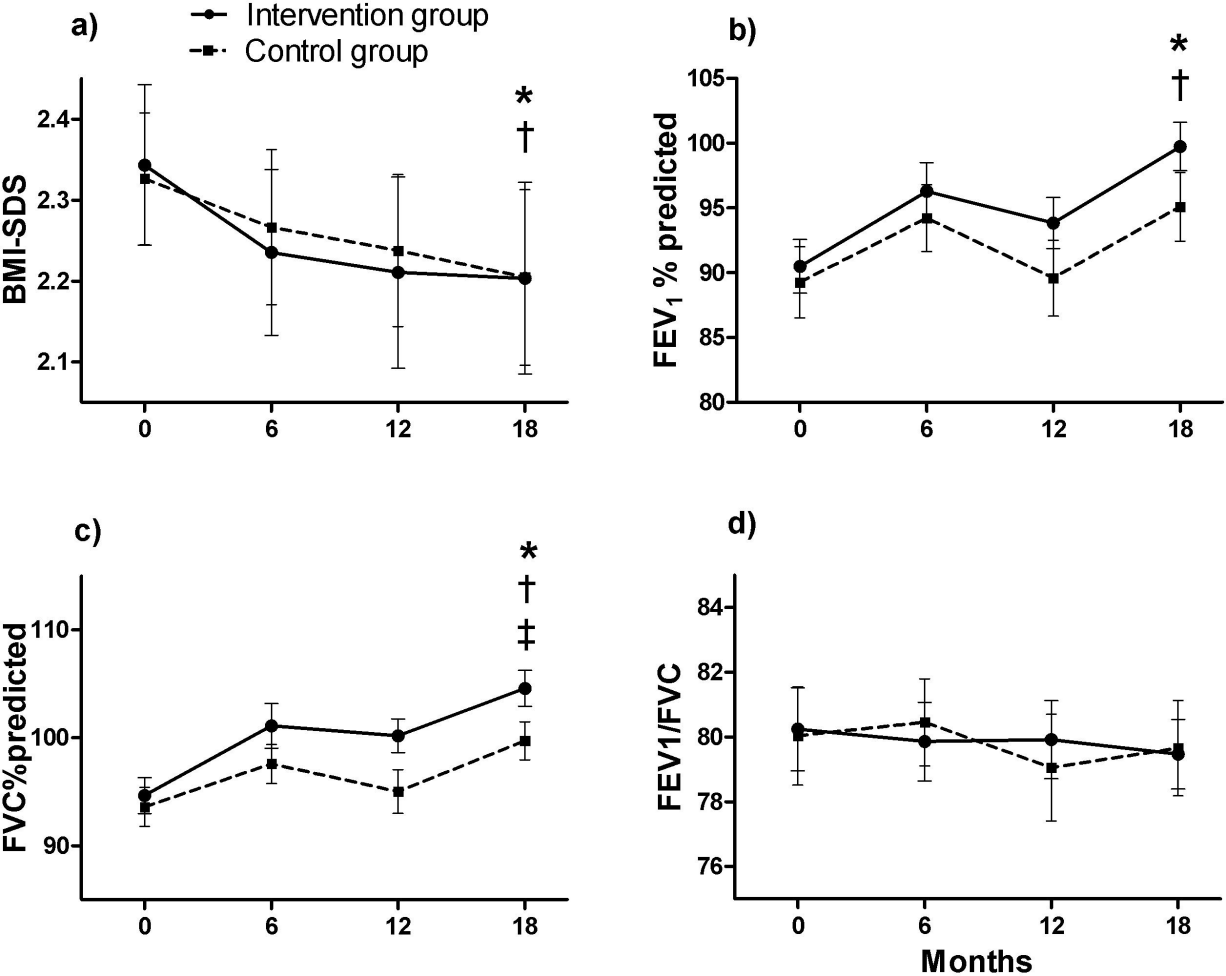
**Changes over time in: a) BMI-SDS, b) FEV**_**1**_**%predicted, c) FVC%predicted, d), FEV1/FVC (in %) §.** Data are presented as mean (SEM). *: Significant difference over time in the intervention group (p<0.05). †: Significant difference over time in the control group (p<0.05). ‡: Significant difference between intervention and control group over time (p<0.05). §: Intention to treat analyses are presented. Abbreviations: BMI-SDS: body mass index standard deviation score, FEV_1_%predicted: forced expiratory volume in 1 second in %predicted, FVC: forced vital capacity in %predicted.

There was no effect of group allocation on the decrease in BMI-SDS, either in terms of the intention to treat (p = 0.84) or per protocol analysis (Fig 2A, p = 0.65). BMI-SDS decreased after the 18-month period by -0.14 ± 0.29 points (Fig 2A, p<0.01) in the intervention group, and -0.12 ± 0.34 points (Fig 2A, p<0.01) in the control group. The changes over time in FEV_1_% predicted were not significantly different between the intervention and control group (Fig 2B, p≥0.05). FEV_1_ improved significantly after 18 months from 90.5 ± 12.5 to 99.7 ± 11.0 (Fig 2B, p<0.05) % predicted in the intervention group, and from 89.3 ± 17.1 to 95.1 ± 16.6 (Fig 2B, p<0.05) % predicted in the control group.

### Secondary research question: other asthma parameters (Figs 2, 3 and 4)

**Fig 3:**
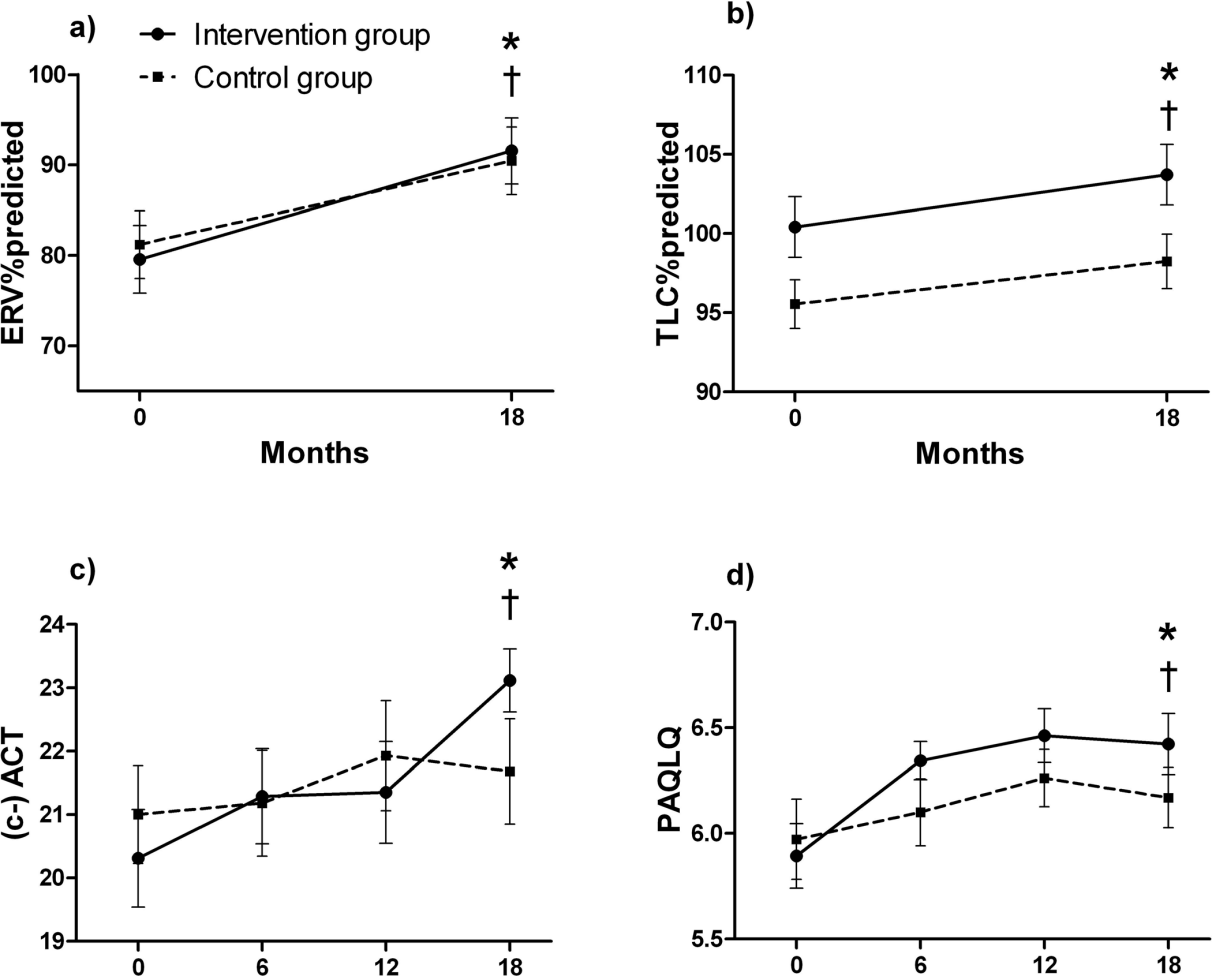
**Changes over time in: a) ERV%predicted, b) TLC%predicted, c) (c)- ACT ll, d) PAQLQ ll §.** Data are presented as mean (SEM). *: Significant difference over time in the intervention group (p<0.05). †: Significant difference over time in the control group (p<0.05). ‡: Significant difference between intervention and control group over time (p<0.05). §: Intention to treat analyses are presented. ll: Only subjects with an asthma diagnosis are shown. Abbreviations: (c)-ACT: (childhood) asthma control test (score can range between 0 = not well controlled to 27 = well controlled asthma), ERV%predicted: expiratory reserve volume in % predicted, PAQLQ, paediatric asthma quality of life questionnaire (with 23 questions in three domains (symptoms, activity limitation and emotional function), all questions can be scored on a 7-point scale (7 = not bothered at all—1 = extremely bothered). The overall PAQLQ score is the mean of all 23 responses.), TLC%predicted: total lung capacity in % predicted.

**Fig 4:**
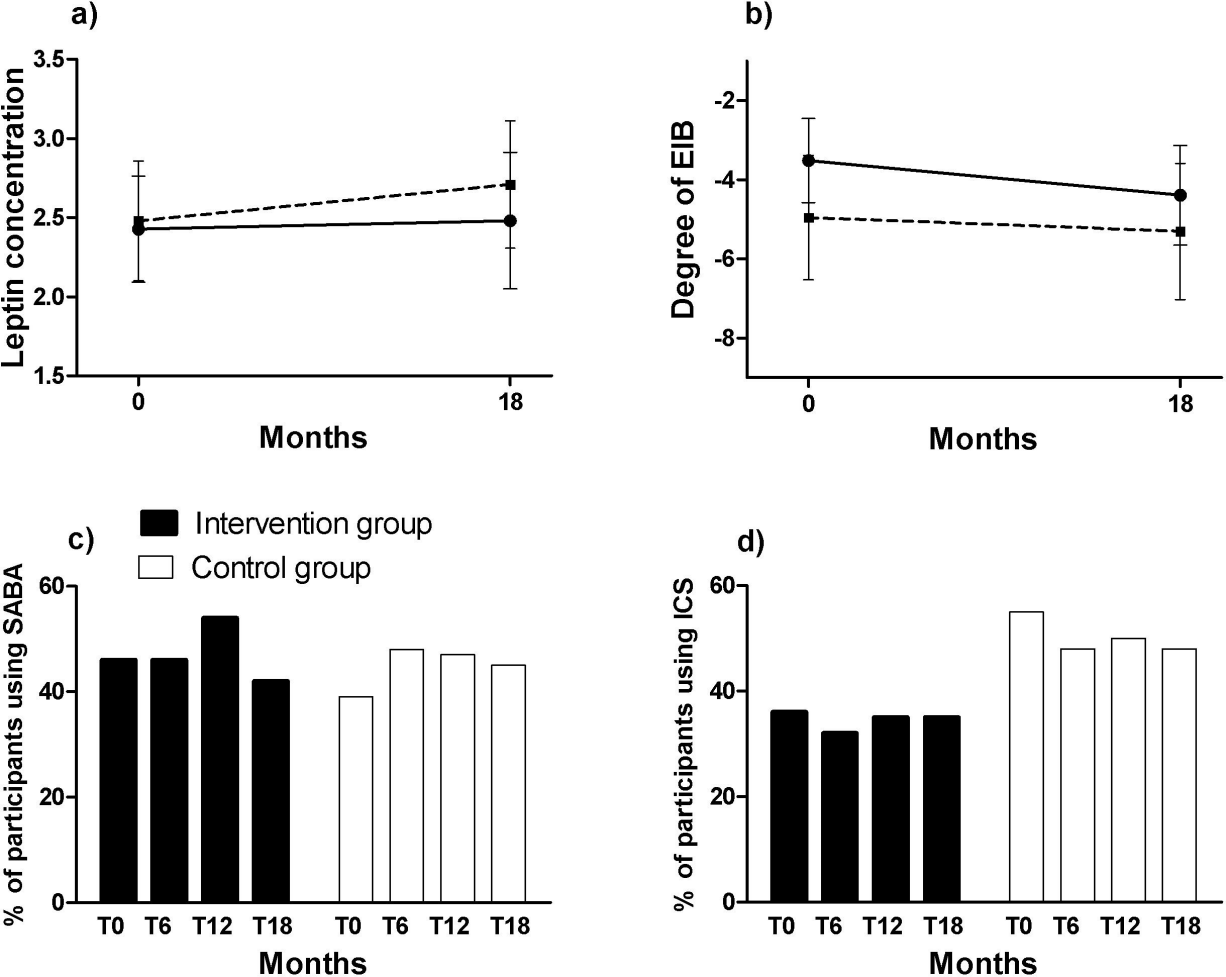
**Changes over time in: a) Degree of EIB (in percent fall of FEV1 after exercise), b) Leptin concentration (in ng/ml), c) % of participants using SABA ll, d) % of participants using ICS ll §.** Data are presented as mean (SEM). *: Significant difference over time in the intervention group (p<0.05). †: Significant difference over time in the control group (p<0.05). ‡: Significant difference between intervention and control group over time (p<0.05). §: Intention to treat analyses are presented. ll: Only subjects with an asthma diagnosis are shown. Abbreviations: EIB: exercise induced bronchoconstriction (in percent fall of FEV1 after exercise test), ICS: inhaled corticosteroids, SABA: short acting beta2agonists.

The improvement in FVC% predicted was significantly greater in the intervention group compared to the control group (Fig 2C, p<0.05). None of the changes in other lung function indices differed between the intervention and control group (Figs 2D, 3A–3C and 4B, p>0.05). FVC% predicted improved over time by 10.1 ± 8.7% in the intervention group (Fig 2C, p<0.001) and 6.1 ± 8.4% in the control group (Fig 2C, p<0.05). In the intervention group, ERV% predicted improved by 12.0 ± 20.5% after 18 months (Fig 3A, p<0.01), and TLC% predicted by 4.0 ± 8.8% (Fig 3B, p<0.05). FEV_1_/FVC% predicted, and the degree of EIB remained unchanged during 18 months in the intervention and control group (Figs 2D and 4B, p>0.05).

Changes over time in asthma control, and the number of patients who used medication (either SABA or ICS) were not statistically different between intervention and control group (Figs 3C, 4C and 4D, p>0.05). PAQLQ improved slightly more in the intervention group than the control group, although the improvement was not significantly different among the groups (Fig 3D, p = 0.06). PAQLQ significantly improved by an average of 0.48 ± 0.72 points (p<0.001) in children with asthma in the intervention group. This is a clinically important difference, as an increase of 0.42 is considered clinically relevant [30]. The (c)-ACT score improved significantly in asthmatic children in the intervention group during the course of 18 months (Fig 3C, p<0.05).

No between and within group changes in inflammatory parameters were observed during the 18-month period. Adiponectin levels decreased from (mean (SD)) 11.20 (4.62) to 10.93 (4.72) ng/ml in the intervention group and from 12.02 (3.68) to 11.95 (3.82) ng/ml in the control group (p>0.05 for between and within group changes). Similar findings were found for leptin (Fig 4A, p>0.05).

### Lifestyle parameters in the intervention and control group

None of the trends in lifestyle parameters were significantly different between intervention and control group (for all parameters, p>0.05) (S1 Fig Additional Figures). Aerobic fitness (VO_2_peak% predicted) was the only lifestyle parameter which significantly changed over time (p<0.01) in the intervention group, though it was not significantly different from the control group.

## Discussion

The most important finding of this study was that deteriorations in body weight/BMI-SDS and lung function in children with overweight/obesity and (a high risk of developing) asthma can be curtailed and to some extent reversed. We not only found significant decreases in BMI-SDS, but also observed clinically relevant improvements in several lung function indices (FEV_1_, FVC, ERV, TLC), asthma control, aerobic fitness, and asthma quality of life (PAQLQ). However, in general, improvements were no greater in the intervention than in the control group with some exceptions. FVC% predicted improved by significantly more in the intervention group compared to the control group, and improvements in asthma control and PAQLQ were more pronounced in the intervention group than in the control group. A considerable number of children/parents in the control group (receiving ‘the usual care’) wished to improve their body weight, lung function and/or asthma outcomes (which was one of the motivations to participate in the study), and sought the extra professional help of a dietician, a weight reduction consultant, or online weight reduction advice during the study. Such behaviour is difficult to prevent in a real-life study such as this.

The improvements in BMI in this study were in line with data of a recent meta-analysis of paediatric weight reduction programs by Waters et al, showing the effects of weight reduction programmes on BMI/BMIz of -0.15kg/m2 (95% CI: -0.21 to -0.09) [31]. Some studies in asthma described weight loss with effects ranging from 0.2 BMI-SDS points to an impressive weight reduction of 6 kg/m^2^ in BMI in one study [10–13, 15]. The results of the latter study could be slightly distorted as only per protocol analyses were shown and no control group was used [10]. Unfortunately, we did not achieve more weight reduction in the intervention than the control group of our study for reasons discussed below.

We found many clinically relevant improvements in asthma features and lung function variables (including FEV_1_% predicted, FVC% predicted, asthma control and asthma-related QOL) in both the intervention and control group. The only variable that improved significantly more in the intervention group was FVC% predicted. The improvement in FEV_1_ and FVC in the intervention group was approximately 10% which is substantial and clinically meaningful. Moreover, there was a non-significant trend towards more improvement in asthma-related QOL in the intervention group compared to the control group. Our promising findings of positive effects of weight reduction on asthma parameters are supported by previous (mostly small and non-controlled) studies, that demonstrated small though relevant improvements in asthma-related QOL, asthma control, lung function indices and EIB after weight reduction [10–13].

How can we explain the lack of contrast between the intervention and control group? Usually, children with overweight and obesity do not decrease, but increase their BMI-SDS over time [7]. Most likely, the control group was motivated to lose weight because of the extra attention and perceived awareness of the problem and the participation in the study [31, 32]. This is reflected in the large number of participants who arranged professional help for weight reduction during the study period. It was not possible to blind the study. We did a lot to stimulate intervention adherence in the intervention group, including imbursements for children when they reached their personal goals, additional social bonding activities, and regular evaluations. This was to some extent successful. The average presence for sport, group, parental and individual sessions was good, and the average heart rate for each sport session was 79% of the maximum heart rate. However, 24 participants did not complete the entire intervention (Fig 1), and the majority of the discontinuers (63%) stopped during the initial six months of the intervention. In a follow-up study, we (qualitatively) analysed the reasons for non-compliance in interviews with both children and caregivers. In those interviews, it was often reported that the high intensity of the programme was difficult to combine with daily life routines, despite numerous efforts of the researchers to adjust time schedules. Based on this experience, our advice for future studies is to include the intervention in a daily routine, such as a school environment, to avoid non-compliance.

The question arises as to whether the lung function improvements could be attributed to improvements in BMI-SDS, and/or mechanical and inflammatory changes. We performed a post-hoc analysis (independent of group allocation), to investigate whether changes in asthma features (FEV_1_% predicted, FVC% predicted and asthma control) were associated with changes in BMI-SDS, changes in leptin or changes in a marker of the mechanical pathway (ERV% predicted) (S4 File. Post hoc analyses). We found that changes over time in either BMI-SDS, leptin and ERV% predicted were not related to changes in FEV_1_% predicted, FVC% predicted, or asthma control in the group as a whole. This may be a consequence of the small changes of BMI-SDS in children over time. When analysed cross-sectionally, we detected an association between more severe asthma features (including low FEV_1_% predicted and FVC% predicted), and a high BMI-SDS and low ERV% predicted. Moreover, an association was found between a marker of the mechanical pathway (low ERV% predicted), but not the inflammatory pathway (leptin) with lower lung functions in overweight/obese children. This implies that children with the most severe obesity have altered mechanical markers, and more severe asthma features.

This study has several methodological strengths [16]: 1) This is a weight reduction study in overweight/obese children with asthma with an RCT design; 2) a considerable number of children (n = 87); 3) a long-term intervention spanning 18 months; 4) outcome measures of both the inflammatory and mechanical pathways.

In addition to these strengths, potential limitations can be mentioned. For the study we included both children with asthma as well as children with overweight/obesity and a family history of asthma. The latter group was included as these children are at a high risk of developing asthma and frequently have respiratory symptoms. Therefore we hypothesised that this group could also benefit from a weight loss intervention. However, this heterogeneity in the study population could have diluted the results and might be considered to be a limitation of the study.). However, we believe that the influence on results is limited in our intervention as outcome parameters such as asthma control and PAQLQ were only determined in the children with asthma. For future studies, a more homogeneous patient population or separate analyses (which our sample size did not allow) of these patient categories is advised, though the study in both groups is relevant as children with overweight/obesity with a high risk of developing asthma can also benefit from weight reduction interventions.

What are the clinical implications of this study? It is evident from this study that the trend towards an increase in weight/BMI can be reversed in overweight/obesity children with (a high risk of developing) asthma, although the lack of contrast between the intervention and control group is somewhat disappointing. Decreases in body weight and BMI in the children were accompanied by clinically relevant improvements in all lung function indices, asthma control, aerobic fitness, and asthma-related quality of life. Another point of interest is the underlying mechanism of the asthma-obesity relationship. In our population we reported a weak association between a marker of the mechanical pathway (ERV% predicted) with BMI-SDS, but no association between a marker of the inflammatory pathway (leptin) with BMI-SDS. In order to avoid the trend in the control group that parents/children look for weight reduction measures, a randomized consent design may be more appropriate for future RCTs on this topic [33].

In summary, this 18-month study on weight reduction shows that overweight/obese children with (a high risk of developing) asthma decreased in body weight and BMI-SDS, and demonstrated clinically meaningful improvements in all lung function indices (FEV_1_, FVC, ERV, TLC), asthma control, aerobic fitness, and asthma quality of life (PAQLQ), although improvements were generally no greater in the intervention than in the control group. Only FVC% predicted improved significantly more in the intervention group compared to the control group, and improvements in asthma control and asthma related quality of life were more pronounced in the intervention group than in the control group. We found some indications for the ‘mechanical pathway’ as an underlying mechanism for the improvement in asthma parameters after weight loss.

## Supporting Information

S1 Fig Additional Figures. (DOCX)

S1 File Study protocol. (PDF)

S2 File Statistical analyses. (DOCX)

S3 File CONSORT statement. (DOC)

S4 File Post hoc analyses. (DOCX)

S1 Table Additional baseline characteristics. (DOCX)
