# Correction: A Multifactorial Weight Reduction Programme for Children with Overweight and Asthma: A Randomized Controlled Trial

**DOI:** 10.1371/journal.pone.0181130

**Published:** 2017-07-06

**Authors:** Maartje Willeboordse, Kim D. G. van de Kant, Frans E. S. Tan, Sandra Mulkens, Julia Schellings, Yvonne Crijns, Liesbeth van der Ploeg, Constant P. van Schayck, Edward Dompeling

The citation appears incorrectly. The correct citation is: Willeboordse M, van de Kant KDG, Tan FES, Mulkens S, Schellings J, Crijns Y, et al. (2016) A Multifactorial Weight Reduction Programme for Children with Overweight and Asthma: A Randomized Controlled Trial. PLoS ONE 11(6): e0157158. https://doi.org/10.1371/journal.pone.0157158.
