# Supplementary material for: A Multifactorial Weight Reduction Programme for Children with Overweight and Asthma: A Randomized Controlled Trial
Source: PLoS One. 2016 Jun 13;11(6):e0157158. doi: 10.1371/journal.pone.0157158 (PMC4905647; doi:10.1371/journal.pone.0157158)
Supplement: S1 Fig — (DOCX) [file pone.0157158.s001.docx]

**S1 FIG: ADDITIONAL FIGURES**

**Change over time in a) Step count, b) Diet score, c) VO_2_peak%predicted, d) DEBQ emotional eating score, e) DEBQ external eating score, f) DEBQ restraint eating score.** §


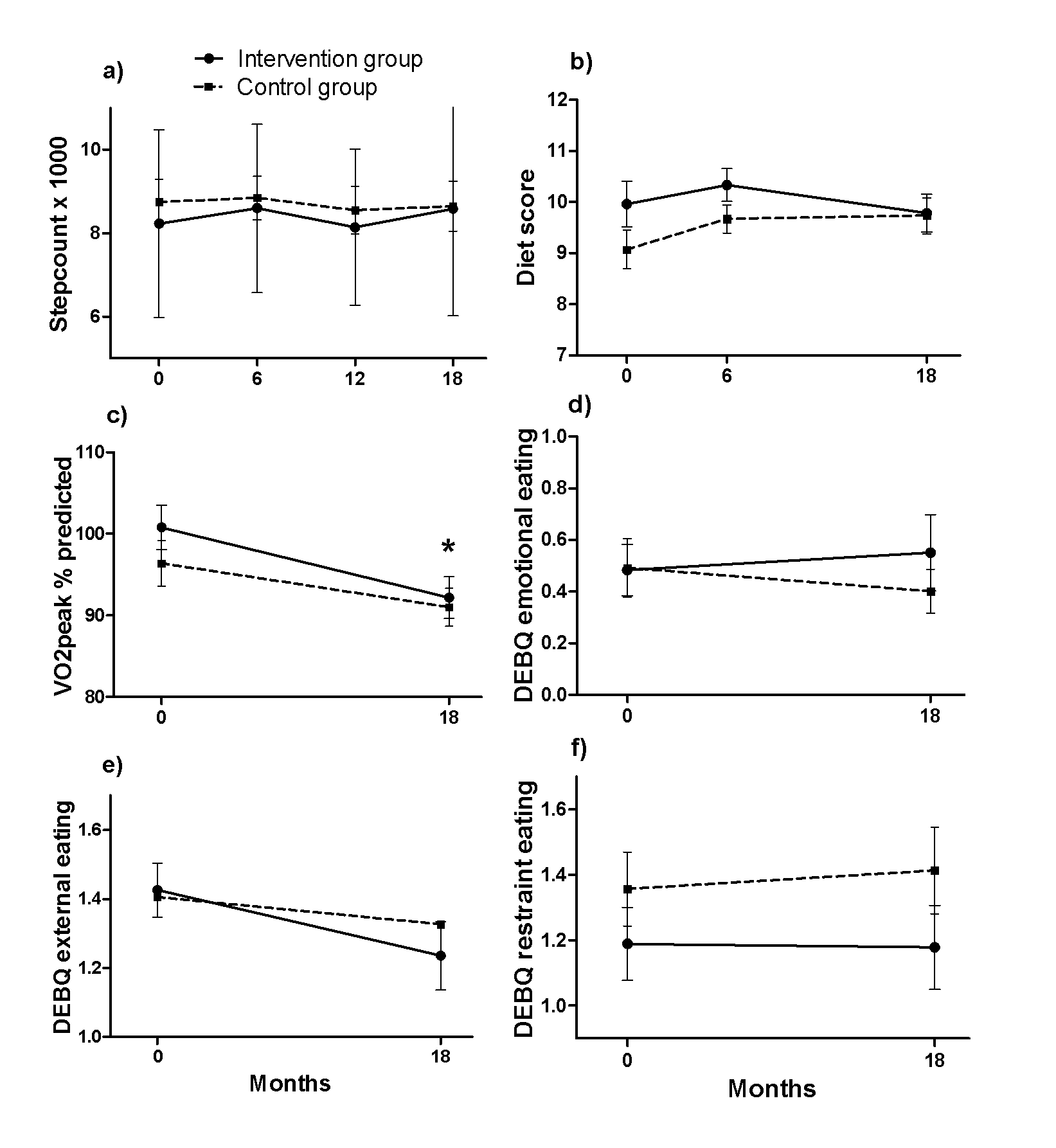


Legend:

Data are presented as mean (SEM).

*: Significant difference over time in the intervention group (p<0.05)

†: Significant difference over time in the control group (p<0.05)

‡: Significant difference between intervention and control group over time (p<0.05)

§: Intention to treat analyses are shown

Abbreviations: DEBQ: dutch eating behaviour questionnaire; VO2peak%predicted: aerobic capacity.
