# Supplementary material for: A Multifactorial Weight Reduction Programme for Children with Overweight and Asthma: A Randomized Controlled Trial
Source: PLoS One. 2016 Jun 13;11(6):e0157158. doi: 10.1371/journal.pone.0157158 (PMC4905647; doi:10.1371/journal.pone.0157158)
Supplement: S1 File — (PDF) [file pone.0157158.s002.pdf]

Willeboordse et al. BMC Public Health 2013, 13:494
http://www.biomedcentral.com/1471-2458/13/494

BMC Public Health

STUDY PROTOCOL Open Access

Multifactorial intervention for children with asthma and overweight (Mikado): study design of a randomised controlled trial

Maartje Willeboordse[1*], Kim DG van de Kant[1], Maroeska N de Laat[1], Onno CP van Schayck[2], Sandra Mulkens[3] and Edward Dompeling[1]

Abstract

Background: In children, the prevalence's of both obesity and asthma are disconcertingly high. Asthmatic children with obesity are characterised by less asthma control and a high need for asthma medication. As the obese asthmatic child is becoming more common in the clinical setting and the disease burden of the asthma-obesity phenotype is high, there is an increasing need for effective treatment in these children. In adults, weight reduction resulted in improved lung function, better asthma control and less need for asthma medication. In children this is hardly studied. The Mikado study aims to evaluate the effectiveness of a long term multifactorial weight reduction intervention, on asthma characteristics in children with asthma and a high body weight.

Methods/design: The Mikado study is a two-armed, randomised controlled trial. In total, 104 participants will be recruited via online questionnaires, pulmonary paediatricians, the youth department of the Municipal Health Services and cohorts of existing studies. All participants will be aged 6–16 years, will have current asthma, a Body Mass Index in the overweight or obesity range, and no serious comorbidities (such as diabetes, heart diseases). Participants in the intervention arm will receive a multifactorial intervention of 18 months consisting of sessions concerning sports, parental involvement, individual counselling and lifestyle advices including dietary advices and cognitive behavioural therapy. The control group will receive usual care. The primary outcome variables will include Forced Expiratory Volume in one second and Body Mass Index - Standard Deviation Score. Secondary outcomes will include other lung function parameters (including dynamic and static lung function parameters), asthma control, asthma-specific quality of life, use of asthma medication and markers of systemic inflammation and airway inflammation.

Discussion: In this randomised controlled trial we will study the potential of a multifactorial weight reduction intervention to improve asthma-related outcome measures in asthmatic children with overweight. Moreover, it will provide information about the underlying mechanisms in the relationship between asthma and a high body weight in children. These findings can contribute to optimal management programs and better clinical guidelines for children with asthma and overweight.

Trial registration: Clinicaltrial.gov NCT00998413

Keywords: Asthmatic, BMI, Child, Obesity, Paediatric, Weight loss

* Correspondence: m.willeboordse@mumc.nl
[1]Department of Paediatric Respiratory Medicine, School for Public Health and Primary Care (CAPHRI), Maastricht University Medical Centre (MUMC), P. Debyelaan 25, 6202, AZ, P.O. Box 5800, Maastricht, The Netherlands
Full list of author information is available at the end of the article

BioMed Central

© 2013 Willeboordse et al.; licensee BioMed Central Ltd. This is an Open Access article distributed under the terms of the Creative Commons Attribution License (http://creativecommons.org/licenses/by/2.0), which permits unrestricted use, distribution, and reproduction in any medium, provided the original work is properly cited.

Background

The increase in childhood obesity in recent decades is a worldwide problem. The global prevalence of overweight and obesity in children increased from 4.2% in 1990 to 6.7% in 2010 [1]. Similar to childhood obesity, the prevalence of childhood asthma has been rising in the previous decades [2]. Asthma is a common chronic disease in children, and accounts for many disability-adjusted life years and considerable medical costs [3].

Several studies demonstrated an association between obesity and childhood asthma [4,5]. A recent meta-analysis by Chen et al. showed a dose-responsiveness of elevated BMI on asthma incidence, expressed as a relative risk (RR) of 1.19 (95%CI: 1.03-1.37) for overweight and a RR of 2.02 (95%CI: 1.16-3.50) for obesity to develop future asthma [5]. Furthermore, obesity is associated with less asthma control and more use of oral corticosteroids [6,7].

Various potential pathophysiological theories have been proposed to explain the association between obesity and asthma. The first theory supports the mechanical effect of obesity on asthma, as obesity can adversely impact lung volumes. It is associated with reductions in Vital Capacity (VC), Forced Expiratory Volume in 1 second (FEV_1) and Expiratory Reserve Volume (ERV) [8,9]. Reduced lung volumes in obesity may result in reduced peripheral airway diameter, which subsequently may disturb smooth muscle function and potentially increase airway obstruction and Bronchial Hyper-Responsiveness (BHR) [8]. A second theory that has received growing attention is the effect of obesity on asthma via inflammatory pathways [7,10]. The chronic inflammatory process created by excess adiposity has been implicated as being an underlying factor in asthma pathogenesis. An increase in fat mass is associated with an increase in systemic inflammatory mediators which can exacerbate airway inflammation via inflammatory mediators such as leptin, adiponectin, Interleukin-6 (IL-6) and Tumour Necrosis Factor-alpha (TNF-α) [7,10]. Several studies noted that the pro-inflammatory effects of serum leptin can modulate airway inflammation [7,11,12].

In addition to those theories, multiple factors are studied which possibly play a role in the asthma-obesity relationship such as dietary factors, a sedentary lifestyle, genetic predisposition, metabolic abnormalities and associated comorbidities such as gastro-oesophageal reflux disease and sleep apnoea [4]. It is most likely that these influential factors and theories are not mutually exclusive, but altogether explain the relationship between asthma and obesity [12].

As obesity has a major impact on several asthma parameters, the effect of weight reduction in obese asthmatic children could be of great value for current treatment guidelines. Studies in adults concerning weight loss and its effect on asthma are promising [13-15]. Significant improvements have been found on asthma control, lung function, exacerbations and use of asthma medication after weight loss [14]. In children, the effects of weight loss have been hardly studied. As the obese asthmatic child is becoming more common in the clinical setting, there is need for a well-designed randomised controlled trial (RCT) in which the effects of weight reduction on asthma in children are studied.

Objective and hypothesis

The Mikado study is an RCT in which the effects of an 18-month multifactorial intervention, on several asthma parameters and anthropometric outcomes in children with asthma and overweight are studied. We hypothesise that a multifactorial intervention will decrease the Body Mass Index –Standard Deviation Score (BMI-SDS) of children with asthma and overweight and will improve asthma related parameters including lung function, airway inflammation, asthma control and the use of asthma medication.

The primary research question is

What are the effects of an 18-month multifactorial intervention on FEV_1%predicted and BMI-SDS?

The secondary research questions are

- What is the effect of a multifactorial intervention on the severity and control of asthma as indicated by lung function (FEV_1/FVC, ERV), asthma symptoms, asthma control, asthma-related quality of life, Exercise Induced Bronchoconstriction (EIB), use of asthma medication and exacerbations?
- What are the underlying mechanisms? Is the intervention accompanied by decreased levels of adipokines and diminished airway inflammation? Can we detect a beneficial effect on airway mechanics/lung function?
- What are the potential moderators and mediators of the intervention effects (e.g. anthropometric changes, changes in physical activity and dietary behaviour, socio economic status, comorbidities and smoking exposure)?

Methods/design

Study design

The study design is an RCT for children with overweight and asthma (Figure 1). Children aged 6–16 years living in Southern Limburg, the Netherlands will be recruited. After a screening visit, participants will be randomised into an intervention group or a control group (Figure 1). The intervention group will receive an intervention lasting for 18 months (see details below), while the control group will receive standard usual care according to the standards of the Dutch Society of General Practitioners

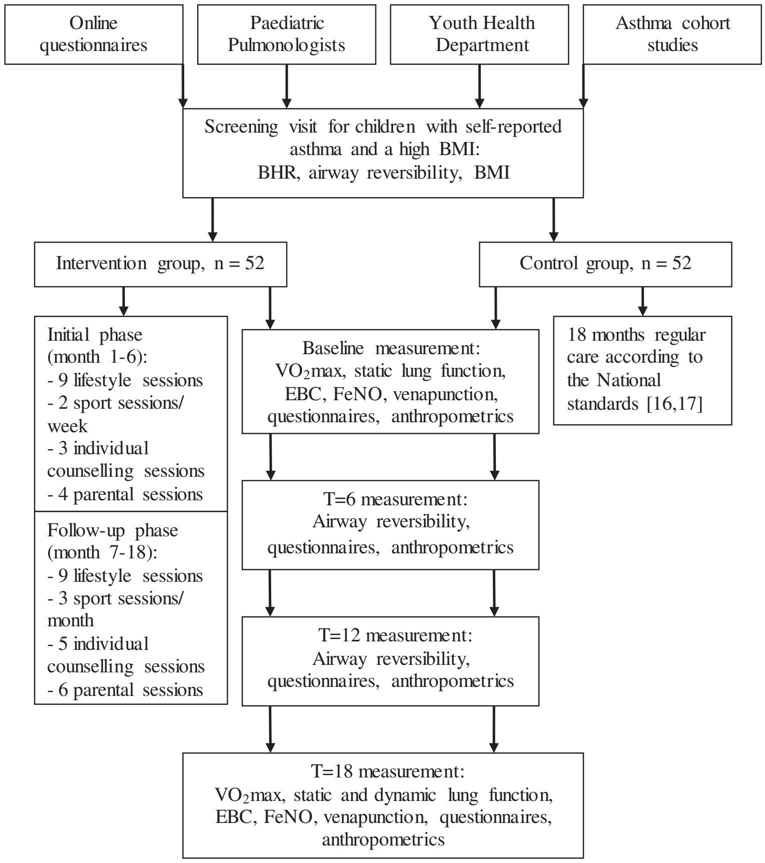

Figure 1 Flow diagram study design. Abbreviations: BHR = Bronchial Hyper-Responsiveness, BMI = Body Mass index, EBC = Exhaled Breath Condensate, FeNO = Fractional exhaled Nitric Oxide, VO_{2max} = Maximal Oxygen Uptake.

and the Paediatric Pulmonology section of the Dutch Society of Paediatrics [16,17].

Regular measurements including lung function outcomes, anthropometric outcomes, inflammatory outcomes and intervention mediators and moderators will be performed at baseline (T=0), after 6 months (T=6), 12 months (T=12) and at the end of the intervention (T=18). Every 3 months, participants will receive various questionnaires (Table 1). All procedures and materials are approved by the Medical Ethic Committee of Maastricht (MEC 09-2-088).

Recruitment

Children will be recruited from 4 sources. The first source is a community-based random online questionnaire to parents of 40,000 children in Southern Limburg. The second source includes pulmonary paediatricians of 3 local hospitals. Thirdly, children are recruited via the youth section of the Municipal Health Services in Southern Limburg. Additionally, children are recruited via cohorts of several other studies [18,19].

All parents will be asked to fulfil an online questionnaire about anthropometric values and asthma symptoms. Children who are overweight according to the parental reported BMI and who reported current asthmatic symptoms and/or use of asthma medication will be invited for a screening visit. Written informed consent will be obtained from all parents and from children aged 12 years or older.

Eligibility criteria

All children with asthma and overweight, aged 6–16 year, living in Southern Limburg will be invited to participate. Overweight will be defined as a BMI-SDS >1 according to the Lambda Mu and Sigma (LMS) method of Cole et al. [20] based on the reference charts of the Dutch fourth Nationwide Growth Study [21]. During the screening visit, participants will undergo lung function measurements to verify the diagnosis of asthma. A child will be considered asthmatic if at least 2 of the 3 following criteria are fulfilled: 1. asthmatic symptoms in the previous 12 months based on the International Study of Asthma and Allergy in childhood (ISAAC) questionnaire [22]; 2. use of Short Acting Beta2agonists (SABA), Long Acting Beta2agonists (LABA) or Inhaled Corticosteroids (ICS) in the previous 6 months; 3. reversibility; defined as an

Table 1 Overview of measurements per visit

	Baseline	T3	T6	T9	T12	T15	T18
**Lung function parameters**							
MEFV curves: FEV_1, FVC, FEV_1/FVC, PEF, MEF_{50} (absolute and %predicted values)*	X		X		X		X
Static lung function test: TLC, RV, FRC, ERV	X						X
BHR	X						
Maximum Ergometry test: VO_{2max}, HR_{max}, %fall in FEV_1	X						X
**Inflammatory parameters**							
FeNO	X						X
EBC: pH, IL-1α, 2, -4, -5, -10, -13, TNF-α, sICAM-1	X						X
Serum: leptin	X						X
**Questionnaires**							
PAQLQ verbal questionnaire for 6–11 year, written questionnaire for 12–16 year	X		X		X		X
EQ-5D-Youth questionnaire	X	X	X	X	X	X	X
c-ACT questionnaire for 6–11 year, ACT questionnaire for 12–16 year	X		X		X		X
ISAAC questionnaire + additional questions about symptoms and exacerbations. Parental version for 6–11 year, self-administered version for 12–16 year	X		X		X		X
GERD questionnaire. Parental version for 6–11 year, self-administered version for 12–16 year	X	X	X	X	X	X	X
PSQ questionnaire. Parental version for 6–11 year, self-administered version for 12–16 year	X		X		X		X
c-DEBQ questionnaire version for 6–11 year, DEBQ for 12–16 year	X						X
SDQ questionnaire. Parental version for 6–11 year, parental and self-administered version for 12–16 year					X		
Demographic factors: e.g. smoking, pets, parental educational level and BMI	X						X
Medication use	X	X	X	X	X	X	X
Health care utilization	X	X	X	X	X	X	X
**Anthropometric values**							
Weight	X	X^i	X	X^i	X	X^i	X
Length	X	X^i	X	X^i	X	X^i	X
Waist and hip circumference	X		X		X		X
4-fold Skinfold measurement	X		X		X		X
**Intervention outcome measures**							
3 × 24 hour food record	X	X^i	X		X		X
7 days accelerometer output	X	X^i	X		X		X
**Continuously measured parameters**							
Percentage presence during the sport sessions, lifestyle sessions, parental sessions and individual counselling sessions. Average heart beat during the sport sessions, BORG score of fatigue [59] after the sport sessions, motivation of the participants (1–10) rated by the sport instructor after the sport sessions, amount of asthma attacks during sporting and safety of the participants.							

Legend: * Pre- and post-bronchodilator spirometry will be obtained during every visit to determine reversibility. At baseline, reversibility will be determined after the BHR test. X^i = Data only obtained in the intervention group.
Abbreviations: *ACT*, Asthma control test; *BHR*, Bronchial hyper-responsiveness; *BMI*, Body mass index; *BORG*, Perceived exertion and pain scale; *c-ACT*, Childhood asthma control test; *c-DEBQ*, Childhood dutch eating behavioural questionnaire; *DEBQ*, Dutch eating behavioural questionnaire; *ERV*, Expiratory reserve volume; *EQ-5D-Youth*, Youth version of Euroqol 5D quality of life questionnaire; *FEV_1*, Forced expiratory volume in 1 second; *FRC*, Functional residual capacity; *FVC*, Forced vital capacity; *GERD*, Gastro oesophageal reflux disease; *HRmax*, Maximum heart rate; *IL*, Interleukin; *ISAAC*, International study of asthma and allergy in childhood; *MEF_{50}*, Maximum expiratory flow rate at 50% of vital capacity; *PAQLQ*, Paediatric asthma quality of life questionnaire; *PEF*, Peak expiratory flow, *pH*, Acidity; *PSQ*, Paediatric sleep questionnaire; *RV*, Residual volume; *SDQ*, Strengths and difficulties questionnaire; *sICAM-1*, Soluble intercellular adhesion molecule-1; *TLC*, Total lung capacity; *TNF-α*, Tumour necrosis factor-alpha; *VO_{2max}*, Maximal oxygen uptake.

increase of ≥9% of the predictive value of the FEV_1 after inhalation of 400 μg Airomir (Teva Pharma, Leiden, the Netherlands) or a positive BHR test; defined as a histamine concentration of ≤8 mg/ml necessary to provoke a drop of 20% in FEV_1.

Children will be excluded in the case of a congenital malformation of the airways or other chronic lung diseases, syndromes accompanied by mental retardation or metabolic diseases, physical limitations to exercise, and/or a heart disease.

Randomisation

All children who meet the inclusion criteria after the screening visit will be randomised to either the control or intervention group. Generalised block randomisation including 10 participants per block will be performed by a computer program, with an allocation ratio of 1:1 [23]. Because this is a lifestyle study, blinding is not possible.

Outcome measures

An overview of all the outcome measurements and corresponding time points can be found in Table 1.

Lung function measurements

Maximal Expiratory Flow Volume (MEFV) curves will be measured with a spirometer (ZAN Messgerate, Oberthulba, Germany) according to American Thoracic Society / European Respiratory Society (ATS/ERS) guidelines [24]. LABA will be withheld 48 hours and SABA 8 hours before the measurement. During these measurements all children will be instructed by experienced researchers and nurses specialised in lung function measurements. The highest value of 3 technically appropriate measurements will be recorded and predictive values will be calculated according to prediction formulas of Zapletal et al. [25]. Reversibility will be defined as a change of ≥9% in FEV_1 after 400 μg of extra-fine salbutamol (Airomir, Teva Pharma, Leiden, the Netherlands). During T0 reversibility will be determined after the completion of the BHR test.

Static lung function indices will be determined by body plethysmography (Viasys, Hoechberg, Germany) according to the ATS/ERS guidelines [24]. BHR will be determined by a histamine provocation test. A dose of histamine will be increasingly administered until a drop of 20% of the FEV_1 is obtained or until a dose of 16 mg/ml histamine is reached [26]. The provocative concentration that will lead to a drop of 20% of the FEV_1 (PC20) will be determined by linear interpolation of the last 2 points on the log dose–response curve.

Anthropometric measurements

Children will be weighed while wearing underwear and without shoes. Length will be measured twice to the nearest 0.5 centimetre (Stadiometer model 213, Seca, Birmingham, United Kingdom). Weight will be measured twice to the nearest 0.1 kilograms using a medical calibrated weight scale (Model 877, Seca, Hamburg, Germany). The average weight and height measurement will be used to calculate the BMI. BMI-SDS scores will be defined by the LMS-method of Cole et. al [20] based on the reference charts of the Dutch fourth Nationwide Growth Study [21].

Skinfold thickness will be derived from the triceps, biceps, subscapular and supra iliacic skinfolds by a Skinfold Calliper (Harpenden, British Indicators, Sussex, United Kingdom). Fat percentages will be generated by using the formulas of Deurenberg et al. [27]. Waist-hip ratio will be obtained by measuring the hip and waist circumference for each visit. Both skin fold thickness and waist-hip circumference measurements will be performed twice according to international guidelines [28].

Maximal ergometry test

All patients will perform a maximal incremental ergometer test (Reha Ergometer, Ergoline, Bitz, Germany) by using a continuous ramp protocol [29]. LABA will be withheld 48 hours and SABA 8 hours before the maximal ergometry test. During the test, breath by breath gas exchange will be analysed by a mass spectrometer (Oxicon Pro, Carefusion, Hochberg, Germany), and Heart Rate (HR) by means of a continuous 12-lead electrocardiogram (Masterscreen ECG, Carefusion, Hochberg, Germany).

The pedalling frequency will be held at 60 rpm and participants will be encouraged during the test to continue as long as possible. At the start, children will rest for 3 minutes in seated position followed by 3 minutes of cycling at the first increment, which is determined by length. Children below 120 cm will undergo a protocol with 10 W/min increment, children 120–150 cm will undergo a protocol with 15 W/min and a 20 W/min increment will be used in children >150 cm.

During the test, the wattage will incrementally increase until children can no longer continue the pedalling frequency for at least 5 seconds, or if termination is required according to other standard safety criteria. After exhaustion, children will continue to cycle for 3 more minutes at the first increment. The test will be accepted if at least 2 of the following criteria will be reached: 1. <2 ml/kg/min increase in oxygen uptake (VO_2) with increasing work load, 2. Respiratory Exchange Ratio > 1.00, 3. HR ≥85%predicted (as assessed by 220 minus age). During the test the following variables will be measured: HR, Minute ventilation (V'E) and VO_2 in L/min and in L/min/kg bodyweight. The Anaerobic Threshold will be determined by the V-slope method as described by Beaver et al. [30], predicted values will be calculated according to normal values of Ten Harkel et al. [31].

MEFV curves will be determined by a spirometer (ZAN Messgerate, Oberthulba, Germany) before exercise, 10 and 30 minutes after completion of the maximal exercise test. The severity of EIB will be measured as the difference in pre-exercise and post-exercise FEV_1.

Questionnaires

Information on socio-demographic characteristics such as age, sex, ethnicity, parental BMI, social economic status and smoking exposure will be collected. Asthma

control will be measured by the Asthma Control Test (ACT) in children >11 years [32]. The childhood ACT (C-ACT) will be used in children ≤11 years [32,33]. A score of ≤19 will be defined as uncontrolled asthma [34]. Medication use will be evaluated over the 2 months period prior to the clinic visit and dose equivalents will be calculated according to standard dosage of SABA, LABA and ICS [16,17,35,36]. The GINA guidelines will be used to determine asthma severity (intermittent, mild, moderate and severe) based on the intensity of treatment [37]. A Dutch questionnaire and the ISAAC questionnaire will be used to measure asthma symptoms [38]. Asthma-related quality of life will be measured by the Paediatric Asthma Quality of Life Questionnaire (PAQLQ) [39]. According to the PAQLQ guidelines, the minimal important difference will be defined as a difference of 0.42 points [40]. The Euroqol 5D Youth version will be used as a measurement tool for general health related quality of life [41].

To investigate commonly associated morbidity in children with asthma, sleep related breathing disorders will be assessed by means of the Paediatric Sleep Questionnaire (PSQ) [42]. A score of >0.33 will be defined as having a high risk for developing a sleep-related breathing disorder [42]. The existence of Gastro-Oesophageal Reflux Disease (GERD) symptoms will be assessed by the GERD questionnaire [43]. A suspicion of GERD is present when the score is ≥ 3 points [43]. Psychosocial problems will be determined by an abnormal score on the Dutch version of the Strength and Difficulties Questionnaire (SDQ) according to the SDQ scoring guidelines [44]. In addition, the Dutch Eating Behaviour Questionnaire (DEBQ) will be used to determine whether children have high scores on the subscales: Emotional Eating (13 items), External Eating (10 items) or Restrained Eating (10 items) [45].

Atopy, systemic and airway inflammation

Systemic inflammation will be determined in blood serum. The serum concentration of leptin will be determined in 100 microliter of plasma by means of multiplex immunoassay (Luminex Corporation, Austin, TX, USA). Airway inflammation will be measured by means of Fractional exhaled Nitric Oxide (FeNO) and markers in Exhaled Breath Condensate (EBC). FeNO will be obtained with the online NIOX analyser (Aerocrine, Solna, Sweden) according to international guidelines [46]. EBC will be collected by means of an optimised glass tube, cooled by countercurrent circulating ice water as described previously [18]. In short, children will breathe tidally into the cooled glass tube for 10 minutes, while wearing a nose-clip, through a mouthpiece connected to a 2-way non-rebreathing valve. Subsequently, acidity will be measured and the EBC samples will be frozen by using dry ice and stored at −80°C. Levels of various cytokine, chemokines, and soluble intercellular adhesion molecule-1 (sICAM-1) will be measured in 100 microliter EBC with multiplex immunoassay (Luminex, Luminex Corporation, Austin, USA) [47].

Physical activity and dietary behaviour

Physical activity level will be measured as the average step count per day measured over a week. All children will be instructed to wear a triaxial accelerometer (Yamax EX510 Power Walker, Yamax, Tokyo, Japan) for 7 consecutive days. While wearing the accelerometer, all children will keep a diary about the time spent on swimming and cycling.

Dietary intake will be measured by means of 3-day food records. Parents and children will receive a standardised instruction based on the multiple-pass method about fulfilling dietary records at the start of the study [48]. An online program (Vodiweb, Vodisys Medical Software, Groningen, The Netherlands) will be used to calculate nutrition composition, based on Western and local food tables and normalised portion sizes [49]. The purpose of this tool is to measure intake of energy and macronutrients (fat, carbohydrate, protein) as well as to differentiate between healthy and unhealthy snacking during the day. A trained researcher will check all food diaries for completeness.

Intervention

We incorporated the most effective components of current weight reduction programs for children in our intervention [50-53]. Several reviews and meta-analyses concluded that long-term (>1 year) multi-component lifestyle interventions targeted at both children and caregivers are most efficient [50-53]. The health counselling model is the theoretical basis of our intervention [54]. The most important components of this intervention are sport sessions, lifestyle sessions including dietary advices and cognitive behavioural therapy, parental sessions, and individualised counselling. Children will be divided in small groups of 8 to 12 children. Intervention group allocation will occur according to place of residence and age (6–11 years and 12–15 years). To secure that intervention professionals adhere to the protocol, all intervention professionals will meet on a regular basis with a study coordinator as supervisor. The intervention is divided in an initial phase (months 0–6) and a follow-up phase (months 7–18) (Figure 1).

Theoretical basis of intervention

The theoretical foundation of the intervention is derived from the health counselling model [54]. Health counselling is a model in which 3 phases for lifestyle changes are included: preparations, behaviour change and follow-up. The initial (preparation and behaviour change) phase will be imbedded during the first 6 months of the intervention in which there are frequent contact moments; the follow-up phase will be imbedded in the last 12 months

(Figure 1). In each phase, well-known behavioural change theories such as the Stages of Change Model, Theory of Planned Behaviour, Social Cognitive Theory and Relapse Prevention Model are imbedded [55-58]. The preparation phase consists of 3 steps: 1. awareness (e.g. unhealthy eating and sedentary behaviour leads to obesity, which leads to health problems and stigmatising), 2. consideration of the new behaviour (e.g. benefits and disadvantages of losing weight) and 3. decision making (e.g. shared decision making including detecting and removing barriers). During the behaviour change phase children will receive more information about healthy behaviour and will be encouraged in each session to set a specific goal which they add to their personal goal list (e.g.: 'I will walk the dog at least 4 days a week for 30 minutes'). In the follow-up phase children will learn how to preserve their behaviour (e.g. creating reminders of their goal-lists in their home environment, avoiding risk situations, learning social and coping skills) and eventually relapse strategies will be made (e.g. making a 'first aid box for difficult situations').

Sport sessions

Sport sessions will consist of regular group exercises (twice a week during initial phase, three times a month during the follow-up phase, Figure 1), with a duration of 60 minutes per session. All sport sessions will be guided by an experienced paediatric physiotherapist or a paediatric sport instructor. A session will consist of 10 minutes warming-up, 20 minutes aerobic exercises, 20–25 minutes interval exercise games and 5 minutes cooling-down. In order to offer an enjoyable program, several recreational sports will be played such as basketball, soccer, rope skipping, and tag games. The duration and intensity of the exercises will gradually increase during the first 3 months of the exercise program. Intensity of the exercise will be held at 60-75% of the age-adjusted maximal HR. All participants will wear a heart rate monitor (polar FS3c, Polar Electro Oy, Kempele, Finland,) during each sport sessions. During and after the sport sessions the (average) HR of each participant will be evaluated by the sports instructor. In addition, Borg's perceived exertion scale is used to monitor exertion [59]. If advised by the children's physician, sport instructors will encourage participants to use SABA 15 minutes before exercise.

During the initial phase participants will be motivated by their sports instructor to choose a supervised sport in their own environment, which children will practice during the follow-up phase. All participants will be encouraged to visit at least 3 different sport clubs in the vicinity of their residence. Folders with sport facilities in the environment will be provided. All parents will be encouraged to support their child to find a new sport during the parental sessions.

Lifestyle sessions

An experienced dietician and psychologist will guide 18 lifestyle sessions with a duration of 75–90 minutes. A session will consist of weighing, evaluation of the previous weeks, personalised goals and BMI-SDS curve, dietary advice usually incorporated in a game, psychological training, personalised goal setting and discussing home work. All children will receive a workbook with additional information for each lifestyle session, homework and space for individualised goal setting. All children will set at least one new personalised goal per session during the initial phase. In the follow-up phase, children will be encouraged to maintain their goals and modify goals in case of no BMI-SDS reduction. Small presents will be provided as incentives for participation and achievements. Individualised incentives will be obtained if personalised goals are reached and group incentives will be obtained in case of high participation rates and preparations for the lifestyle sessions. The dietician and psychologist will put emphasis on positive reinforcement during the lifestyle sessions.

All dietary advices are based on the Dutch dietary guidelines for children with a high body weight [60] and modelled after the work of Dutch programs to prevent childhood obesity, Realfit and Slimkids [61]. During the sessions, children will follow 3 basic dietary guidelines: 1. healthy food choice, 2. regular eating pattern, and 3. normalised portion sizes. Other dietary themes that will be dealt with during the lifestyle sessions are among others: energy balance, fruit and vegetable intake, mindful eating, finding social support, trying new food items (including 2 taste session sessions) inspecting food labels and, if applicable, alcoholic beverages. Special attention will be paid to emotional eating, body dissatisfaction and disordered eating. Also, children will regularly comment on their own and other's dietary journals. If children have not reduced their BMI-SDS after 6 lifestyle sessions, the dietician will provide the children with a personalised balanced hypo caloric diet with low-fat, nutrient dense foods of moderate proportion sizes. The diet will consist of a caloric restriction of 15% less than required [62]. Participants and parents will be guided and encouraged by the dietician to follow the hypo caloric diet and to adjust the diet with the nutritional knowledge they have gained during the previous sessions.

The psychologist will teach cognitive behavioural techniques. Children will learn how to identify, challenge and change dysfunctional cognitions about weight/obesity, food/eating, bullying, self-esteem, sedentary behaviour and physical activity by means of background information and homework assignments including 'thought diaries'. Other themes on which the psychologist will focus are motivation monitoring, stimulus control, recognizing emotions and social skills. Children will be taught how

to cope with real-life situations such as holidays, parties, celebrations, and restaurants. During the follow-up phase a relapse prevention schedule will be made by all participants. The cognitive behaviour protocols were modelled after the work of Werrij et al. [63,64].

The dietician and psychologist will guide participants to decrease sedentary behaviour. Children will be motivated to reach the advised daily 60 minutes of moderate to vigorous physical activity and to perform exhaustive physical activity for 20 minutes at least 3 days per week. Main themes to be discussed are screen time, active transportation ways and daily activity patterns.

Parental sessions

Parents will follow 10 parental sessions of 60 minutes guided by the dietician and psychologist who also guide the lifestyle sessions. A parental session will consist of an evaluation of the prior period, summarising the content of children's lifestyle sessions, dietary or psychological information and goal setting. The parents will receive a workbook including background information, healthy recipes and sport facilities in their residence. The dietary information will consist of standard nutritional education that is also taught to the children. In addition, advices are given about healthy cooking habits, preparing healthy snacks for children and visibility of (un)healthy food at home. The psychologist will focus on parental techniques such as positive rewarding, managing problem behaviours and modelling behaviour of the parents. Emphasis will be put on the importance of individual incentives if children reach their personalised learning goals.

Individual counselling sessions

In addition to the lifestyle sessions, children will receive individualised counselling sessions. The individualised counselling sessions will be guided by either the dietician or the psychologist, dependent on the needs of the child. The individualised counselling sessions will focus on the learning goals, motivational problems, personal barriers for maintaining the leaning goals and possible depressive symptoms. Children aged 6–11 years always attend the individual sessions accompanied by a parent. Older children will be accompanied by a parent if one of the parties (e.g. dietician, psychologist, child or parents) deems a parental visit beneficial. If the child still experiences severe problems with weight reduction despite the regular lifestyle sessions and individual consults, the dietician or psychologist will schedule extra telephonic consults with the child and/or parent.

Statistics

Sample size calculation

Earlier studies on weight loss in asthmatic adults demonstrated improvements in FEV_1 of 8% and 6% respectively [65,66]. In adolescents with asthma, improvements in FEV_1 of up to 24% were found after a weight reduction program [67]. In total, 104 participants are sufficient to detect a change of 5% in FEV_1%, assuming a five-repeated measurement design of Hedeker et al., a two-sided alpha of 5%, a correlation between measurements of 0.7, a power of 80%, a sample size ratio of 1:1, a drop-out rate of 5% and an autoregressive(1) variance-covariance matrix structure [68].

According to the Fleisch method of power calculation, 80 participants are sufficient to detect a clinical relevant decrease of 0.25 BMI-SDS, with a two-sided alpha of 0.05, a drop-out rate of 20% and a power of 90% [69].

Data analysis of primary and secondary research question

Data will be analysed by using Statistical Package for the Social Sciences (version 20.0, SPSS, Chicago, USA). Standard statistical techniques will be used for the baseline characteristics. Normal distributed variables will be expressed as means and standard deviations, non-normal distributed data will be presented as median with interquartile ranges. Multi-level analyses techniques will be used to test whether within-subject changes in FEV_1%predicted after 18 months intervention differ between intervention and control group. Both per-protocol and intention-to-treat-analyses will be performed. The primary outcome FEV_1%predicted will be tested two-tailed with an alpha of 5%. Correction for multiple testing will be conducted for the secondary outcomes.

Discussion

This paper describes the design of the Mikado study, an RCT testing the effects of a multifactorial weight loss intervention on asthma parameters in children with overweight and asthma. The multifactorial intervention consists of sessions concerning diet and behaviour, sport, parental involvement and individual counselling. Emphasis is put on individual goal setting, positive reflections and reinforcements. The efficacy of the Mikado study will be measured by regular anthropometric and lung function measurements. In addition, this study should provide enhanced understanding of the underlying pathophysiology of the asthma-obesity relation in children.

The Mikado study is, up to now, the first RCT to investigate the effects of a weight reduction intervention on asthma parameters in children. Strengths of the Mikado study include its long duration, its broad range of outcome measures and the fact that the intervention is comprised of successful components of previous studies.

There are several critical success factors that should be mentioned. Previous lifestyle studies in children have frequently reported recruitment problems and dropout rates as high as 42% [51,70]. The main reasons for these were parental attitudinal issues, such as denial that their child

would benefit from improved nutrition and exercise, inflexibility in making room in the family's schedule for the program, prohibitive costs and an unwillingness to change the home environment. We will try to minimise these barriers for recruitment by training the researchers in telephonic skills, and by inviting hesitant participants for a personal informative meeting regarding the study. Offering an individualised programme and closely monitoring the participants by the intervention team should minimalize dropout. In case of high dropout rates, we will perform a responsive evaluation to investigate the reasons [71].

Asthma is a chronic disease characterised by regular symptoms, a decreased quality of life, and medication use throughout the entire lifespan. Asthma in childhood has many non-modifiable risk factors such as a family history of asthma, sex and atopy [72]. Obesity is one of the few modifiable risk factors for asthma, which implies the necessity to investigate whether or not weight reduction has the potential to improve asthma-related characteristics. If proven effective, a weight reduction program should be incorporated into the guidelines for childhood asthma. Weight reduction in asthmatic children might lead to an improved asthma prognosis via an improved asthma-related quality of life, asthma control, and lung function.

Abbreviations

ACT: Asthma control test; BHR: Bronchial hyper-responsiveness; BMI: Body mass index; BMI-SDS: Body mass index – standard deviation score; c-ACT: Child version asthma control test; EBC: Exhaled breath condensate; EIB: Exercise induced bronchoconstriction; ERV: Expiratory reserve volume; EQ-5D-youth: Youth version of Euroqol 5D quality of life questionnaire; DEBQ: Dutch eating behaviour checklist; FeNO: Fractional exhaled nitric oxide; FEV_1: Forced expiratory volume in 1 second; FVC: Forced vital capacity; FRC: Functional residual capacity; GERD: Gastro oesophageal reflux disease; HR: Heart rate; ICS: Inhaled corticosteroids; IL: Interleukin; ISAAC: International study of asthma and allergy in childhood; LABA: Long acting Beta2Agonists; MEFV: Maximally expiratory flow volume; MEF50: Maximum expiratory flow rate at 50% of vital capacity; PAQLQ: Paediatric asthma quality of life questionnaire; PC20: The provocative concentration which will lead to a drop of 20% of the FEV_1 after a histamine BHR test; PEF: Peak expiratory flow; pH: Acidity level; PSQ: Paediatric sleep questionnaire; RCT: Randomised controlled trial; RV: Residual volume; SABA: Short acting Beta2Agonists; SDQ: Strengths and difficulties questionnaire; TLC: Total lung capacity; TNF-α: Tumour necrosis factor-alpha; VO2max: Maximal oxygen uptake; VO2peak: Peak oxygen consumption; Wmax: Maximum power.

Competing interests

All authors declare that they have no financial or non-financial competing interests to disclose that are relevant to the implementation of this research and this publication.

Author's contributions

MW is the investigator of the study, has the overall responsibility for the study design and conducted and drafted the manuscript. KK contributed to drafting the manuscript. KK, ED and CS designed the study protocol and revised the manuscript critically for important intellectual content. SM is co-author of the intervention protocol and responsible for critical revision of the cognitive behavioural therapy section and important intellectual content. ML has made a substantial contribution to the conception of the outcome measures, and critically revised the manuscript for intellectual content. All authors read and approved the final document.

Acknowledgements

The project is supported by the Dutch Asthma foundation (NAF 3.4.09.002). We would like to thank the paediatric department of the youth health care centre (GGD South-Limburg), the civil affairs departments of Southern Limburg, all participating paediatric pulmonologists and the lung function and dietary department of the Maastricht University Medical Centre for their cooperation. We wish to thank Vera van Wanrooij, medical student, for her contribution to the Medical Ethic Committee protocol and Anouk Reijnen for her contribution to the intervention protocol.

Author details

[1]Department of Paediatric Respiratory Medicine, School for Public Health and Primary Care (CAPHRI), Maastricht University Medical Centre (MUMC), P. Debyelaan 25, 6202, AZ, P.O. Box 5800, Maastricht, The Netherlands. [2]Department of General Practice, CAPHRI, MUMC, P. Debyelaan 25, 6202, AZ, P.O. Box 5800, Maastricht, The Netherlands. [3]Department of Clinical Psychological Science, Faculty of Psychology and Neuroscience, Maastricht University, P.O. Box 616, 6200, MD Maastricht, The Netherlands.

Received: 13 May 2013 Accepted: 17 May 2013
Published: 21 May 2013

References

1. de Onis M, Blossner M, Borghi E: **Global prevalence and trends of overweight and obesity among preschool children.** *Am J Clin Nutr* 2010, **92**(Suppl 5):1257–1264.
2. Anandan C, Nurmatov U, van Schayck OC, Sheikh A: **Is the prevalence of asthma declining? Systematic review of epidemiological studies.** *Allergy* 2010, **65**(Suppl 2):152–167.
3. Cruz A, Mantzouranis E, Matricardi PM, Minelli E: *Global surveillance, prevention and control of chronic respiratory diseases: a comprehensive approach.* Geneva: WHO press; 2007.
4. Papoutsakis C, Priftis KN, Drakouli M, Prifti S, Konstantaki E, Chondronikola M, Antonogeorgos G, Matziou V: **Childhood overweight/obesity and asthma: is there a link? A systematic review of recent epidemiologic evidence.** *J Acad Nutr Diet* 2013, **113**(Suppl 1):77–105.
5. Chen YC, Dong GH, Lin KC, Lee YL: **Gender difference of childhood overweight and obesity in predicting the risk of incident asthma: a systematic review and meta-analysis.** *Obes Rev* 2012, **13**(Suppl 3):222–231.
6. Lang JE, Hossain J, Smith K, Lima JJ: **Asthma severity, exacerbation risk, and controller treatment burden in underweight and obese children.** *J Asthma* 2012, **49**(Suppl 5):456–463.
7. Jensen ME, Collins CE, Gibson PG, Wood LG: **The obesity phenotype in children with asthma.** *Paediatr Respir Rev* 2011, **12**(Suppl 3):152–159.
8. Jones RL, Nzekwu MM: **The effects of body mass index on lung volumes.** *Chest* 2006, **130**(Suppl 3):827–833.
9. Littleton SW: **Impact of obesity on respiratory function.** *Respirology* 2012, **17**(Suppl 1):43–49.
10. Sin DD, Sutherland ER: **Obesity and the lung: 4. Obesity and asthma.** *Thorax* 2008, **63**(Suppl 11):1018–1023.
11. Shore SA: **Obesity and asthma: possible mechanisms.** *J Allergy Clin Immunol* 2008, **121**(Suppl 5):1087–1093.
12. Farah CS, Salome CM: **Asthma and obesity: a known association but unknown mechanism.** *Respirology* 2012, **17**(Suppl 3):412–421.
13. Eneli IU, Skybo T, Camargo CA Jr: **Weight loss and asthma: a systematic review.** *Thorax* 2008, **63**(Suppl 8):671–676.
14. Juel CT, Ali Z, Nilas L, Ulrik CS: **Asthma and obesity: does weight loss improve asthma control? a systematic review.** *J Asthma Allergy* 2012, **5**:21–26.
15. Adeniyi FB, Young T: **Weight loss interventions for chronic asthma.** *Cochrane Database Syst Rev* 2012, **7**, CD009339.
16. Jongste JC, Vrijlandt ELJE: *Astma bij kinderen; herziene richtlijnen van de sectie kinderlongziekten van de NVK [Asthma in children; revised guidelines of the paediatric department of the Dutch Paediatric Society].* Amsterdam; 2007. ISBN 978-90-9023508-0.
17. Bindels PJE, Van de Wouden JC, Ponsioen BP, Brand PLP, Salome PL, Van Hensbergenm W: **NHG-standaard Astma bij kinderen (tweede herziening) [Dutch General Practitioners guidelines, asthma in children].** *Huisarts en Wetenschap* 2006, **49**(Suppl 11):557–572.
18. van de Kant KD, Klaassen EM, Jobsis Q, Nijhuis AJ, van Schayck OC, Dompeling E: **Early diagnosis of asthma in young children by using non-invasive biomarkers of airway inflammation and early lung function measurements: study protocol of a case–control study.** *BMC Publ Health* 2009, **9**(Suppl 1):210.

19. Schonberger HJ, Dompeling E, Knottnerus JA, Maas T, Muris JW, van Weel C, van Schayck CP: **The PREVASC study: the clinical effect of a multifaceted educational intervention to prevent childhood asthma.** *Eur Respir J* 2005, **25**(Suppl 4):660–670.
20. Cole TJ, Green PJ: **Smoothing reference centile curves: the LMS method and penalized likelihood.** *Stat Med* 1992, **11**(Suppl 10):1305–1319.
21. Fredriks A, van Buuren S, Wit J, Verloove-Vanhorick S: **Body index measurements in 1996–7 compared with 1980.** *Arch Dis Child* 2000, **82:**107–112.
22. Asher MI, Montefort S, Bjorksten B, Lai CK, Strachan DP, Weiland SK, Williams H: **Worldwide time trends in the prevalence of symptoms of asthma, allergic rhinoconjunctivitis, and eczema in childhood: ISAAC phases one and three repeat multicountry cross-sectional surveys.** *Lancet* 2006, **368**(Suppl 9537):733–743.
23. Addelman S: **The generalized randomized block design.** *Am Stat* 1969, **23**(Suppl 4):35–36.
24. Miller MR, Hankinson J, Brusasco V, Burgos F, Casaburi R, Coates A, Crapo R, Enright P, van der Grinten CP, Gustafsson P, *et al*: **Standardisation of spirometry.** *Eur Respir J* 2005, **26**(Suppl 2):319–338.
25. Zapletal A, Paul T, Samánek N: **Die Bedeutung heutiger Methoden der Lungenfunktionsdiagnostik zur Feststellung einer Obstruktion der Atemwege bei Kindern und Jugendlichen.** *Atm-Org* 1977, **149:**343–371.
26. Society AT: **Guidelines for methacholine and exercise challenge testing.** *Am J Respir Crit Care Med* 2000, **161:**309–329.
27. Deurenberg P, Pieters JJ, Hautvast JG: **The assessment of the body fat percentage by skinfold thickness measurements in childhood and young adolescence.** *Br J Nutr* 1990, **63**(Suppl 2):293–303.
28. Marfell-Jones M, Olds T, Stewart AD, Carter L: **International Standards for Anthropometric Assessment**. In *International Society for the Advancement of Kinanthropometry (ISAK).* Potchefstroom, South Africa; 2006.
29. Davis JA, Whipp BJ, Lamarra N, Huntsman DJ, Frank MH, Wasserman K: **Effect of ramp slope on determination of aerobic parameters from the ramp exercise test.** *Med Sci Sports Exerc* 1982, **14**(Suppl 5):339–343.
30. Beaver WL, Wasserman K, Whipp BJ: **A new method for detecting anaerobic threshold by gas exchange.** *J Appl Physiol* 1986, **60**(Suppl 6):2020–2027.
31. Ten Harkel AD, Takken T, Van Osch-Gevers M, Helbing WA: **Normal values for cardiopulmonary exercise testing in children.** *Eur J Cardiovasc Prev Rehabil* 2011, **18**(Suppl 1):48–54.
32. Nathan RA, Sorkness CA, Kosinski M, Schatz M, Li JT, Marcus P, Murray JJ, Pendergraft TB: **Development of the asthma control test: a survey for assessing asthma control.** *J Allergy Clin Immunol* 2004, **113**(Suppl 1):59–65.
33. Liu AH, Zeiger R, Sorkness C, Mahr T, Ostrom N, Burgess S, Rosenzweig JC, Manjunath R: **Development and cross-sectional validation of the childhood asthma control test.** *J Allergy Clin Immunol* 2007, **119**(Suppl 4):817–825.
34. Koolen BB, Pijnenburg MW, Brackel HJ, Landstra AM, van den Berg NJ, Merkus PJ, Hop WC, Vaessen-Verberne AA: **Comparing global initiative for asthma (GINA) criteria with the childhood asthma control test (C-ACT) and asthma control test (ACT).** *Eur Respir J* 2011, **38**(Suppl 3):561–566.
35. Nightingale JA, Rogers DF, Barnes PJ: **Comparison of the effects of salmeterol and formoterol in patients with severe asthma.** *Chest* 2002, **121**(Suppl 5):1401–1406.
36. Wong CS, Pavord ID, Williams J, Britton JR, Tattersfield AE: **Bronchodilator, cardiovascular, and hypokalaemic effects of fenoterol, salbutamol, and terbutaline in asthma.** *Lancet* 1990, **336**(Suppl 8728):1396–1399.
37. Bateman ED, Hurd SS, Barnes PJ, Bousquet J, Drazen JM, FitzGerald M, Gibson P, Ohta K, O'Byrne P, Pedersen SE, *et al*: **Global strategy for asthma management and prevention: GINA executive summary.** *Eur Respir J* 2008, **31**(Suppl 1):143–178.
38. Asher MI, Keil U, Anderson HR, Beasley R, Crane J, Martinez F, Mitchell EA, Pearce N, Sibbald B, Stewart AW: **International study of asthma and allergies in childhood (ISAAC): rationale and methods.** *Eur Respir J* 1995, **8**(Suppl 3):483–491.
39. Juniper EF, Guyatt GH, Feeny DH, Ferrie PJ, Griffith LE, Townsend M: **Measuring quality of life in children with asthma.** *Qual Life Res* 1996, **5**(Suppl 1):35–46.
40. Raat H, Bueving HJ, de Jongste JC, Grol MH, Juniper EF, van der Wouden JC: **Responsiveness, longitudinal- and cross-sectional construct validity of the pediatric asthma quality of life questionnaire (PAQLQ) in dutch children with asthma.** *Qual Life Res* 2005, **14**(Suppl 1):265–272.
41. Wille N, Badia X, Bonsel G, Burstrom K, Cavrini G, Devlin N, Egmar AC, Greiner W, Gusi N, Herdman M, *et al*: **Development of the EQ-5D-Y: a child-friendly version of the EQ-5D.** *Qual Life Res* 2010, **19**(Suppl 6):875–886.
42. Chervin RD, Hedger K, Dillon JE, Pituch KJ: **Pediatric sleep questionnaire (PSQ): validity and reliability of scales for sleep-disordered breathing, snoring, sleepiness, and behavioral problems.** *Sleep Med* 2000, **1**(Suppl 1):21–32.
43. Nelson SP, Chen EH, Syniar GM, Christoffel KK: **Prevalence of symptoms of gastroesophageal reflux during childhood: a pediatric practice-based survey. Pediatric practice research group.** *Arch Pediatr Adolesc Med* 2000, **154**(Suppl 2):150–154.
44. Muris P, Meesters C, van den Berg F: **The strengths and difficulties questionnaire (SDQ)–further evidence for its reliability and validity in a community sample of Dutch children and adolescents.** *Eur Child Adolesc Psychiatry* 2003, **12**(Suppl 1):1–8.
45. van Strien T, Oosterveld P: **The children's DEBQ for assessment of restrained, emotional, and external eating in 7- to 12-year-old children.** *Int J Eat Disord* 2008, **41**(Suppl 1):72–81.
46. Erzurum SC, Deykin A, Dweik R, Laskowski D, Baraldi E, Lundberg JO, George SC, Marczin N, *et al*: **ATS/ERS recommendations for standardized procedures for the online and offline measurement of exhaled lower respiratory nitric oxide and nasal nitric oxide, 2005.** *Am J Respir Crit Care Med* 2005, **171**(Suppl 8):912–930.
47. van de Kant KD, Klaassen EM, Jobsis Q, Koers K, Rijkers GT, van der Grinten CP, van Schayck OC, Passos VL, Dompeling E: **Wheezing in preschool children is associated with increased levels of cytokines/chemokines in exhaled breath condensate.** *J Allergy Clin Immunol* 2010, **126**(Suppl 3):669–671.
48. Moshfegh AJ, Rhodes DG, Baer DJ, Murayi T, Clemens JC, Rumpler WV, Paul DR, Sebastian RS, Kuczynski KJ, Ingwersen LA, *et al*: **The US department of agriculture automated multiple-pass method reduces bias in the collection of energy intakes.** *Am J Clin Nutr* 2008, **88**(Suppl 2):324–332.
49. National Institute for Public Health and the Environment: *NEVO Dutch Food Composition Database online version 2011/3.0.* http://www.rivm.nl/en/Topics/Topics/D/Dutch_Food_Composition_Database/Access_NEVO_data/NEVO_online.
50. McGovern L, Johnson JN, Paulo R, Hettinger A, Singhal V, Kamath C, Erwin PJ, Montori VM: **Clinical review: treatment of pediatric obesity: a systematic review and meta-analysis of randomized trials.** *J Clin Endocrinol Metab* 2008, **93**(Suppl 12):4600–4605.
51. Oude Luttikhuis H, Baur L, Jansen H, Shrewsbury VA, O'Malley C, Stolk RP, Summerbell CD: **Interventions for treating obesity in children.** *Cochrane Database Syst Rev* 2009, **CD001872-CD001872**(Suppl 1):CD001872.
52. Snethen JA, Broome ME, Cashin SE: **Effective weight loss for overweight children: a meta-analysis of intervention studies.** *J Pediatr Nurs* 2006, **21**(Suppl 1):45–56.
53. Waters E, de Silva-Sanigorski A, Hall BJ, Brown T, Campbell KJ, Gao Y, Armstrong R, Prosser L, Summerbell CD: **Interventions for preventing obesity in children.** *Cochrane Database Syst Rev* 2011, **12:**CD001871.
54. Gerards F, Borgers R: *Health counseling; Het adviesgesprek in de (para) medische en verpleegkundige zorg [health counselling; advisory conversations in the (para)medical care and nursery care].* Soest: Uitgeverij Nelissen; 2006.
55. Prochaska JDCC: *The transtheoretical approach.* 2nd edition. New York: Oxford University Press; 2005.
56. Ajzen I: **The theory of planned behavior.** *Organ Behav Hum Decis Process* 1991, **50**(Suppl 2):179–211.
57. Bandura A: **Self-efficacy: toward a unifying theory of behavioral change.** *Psychol Rev* 1977, **84**(Suppl 2):191–215.
58. Marlatt GA, George WH: **Relapse prevention: introduction and overview of the model.** *Br J Addict* 1984, **79**(Suppl 3):261–273.
59. Borg G: *Borg's perceived exertion and pain scales.* Champaigne: Human Kinetics; 1998.
60. Wezel BJ: *Dieetbehandelingsrichtlijnen: Te hoog gewicht bij kinderen en adolescenten (4–18 jaar) [Dietary guidelines: An increased bodyweight in children and adolescents (aged 4–18)].* Maarssen: Elsevier Gezondheidszorg; 2004.
61. Huis voor de sport, Thuiszorg Westelijke Mijnstreek, GGD Westelijke Mijnstreek, Universiteit Maastricht: *Guidebook Realfit, information for professionals.* Sittard; 2005.
62. Schofield WN: **Predicting basal metabolic rate, new standards and review of previous work.** *Hum Nutr Clin Nutr* 1985, **39**(Suppl 1):5–41.
63. Werrij MQ, Jansen A, Mulkens S, Elgersma HJ, Ament AJ, Hospers HJ: **Adding cognitive therapy to dietetic treatment is associated with less relapse in obesity.** *J Psychosom Res* 2009, **67**(Suppl 4):315–324.

64. Mulkens S, Fleuren D, Nederkoorn C, Meijers J: **Realfit: een multidisciplinaire groepsbehandeling voor jongeren met overgewicht [Realfit: a multidisciplinary group treatment program for adolescents with overweight].** *Gedragstherapie* 2007, **40:**27–48.
65. Stenius-Aarniala B, Poussa T, Kvarnstrom J, Gronlund EL, Ylikahri M, Mustajoki P: **Immediate and long term effects of weight reduction in obese people with asthma: randomised controlled study.** *BMJ (Clinical research ed* 2000, **320**(Suppl 7238):827–832.
66. Hakala K, Stenius-Aarniala B, Sovijarvi A: **Effects of weight loss on peak flow variability, airways obstruction, and lung volumes in obese patients with asthma.** *Chest* 2000, **118**(Suppl 5):1315–1321.
67. da Silva PL, de Mello MT, Cheik NC, Sanches PL, Correia FA, de Piano A, Corgosinho FC, Campos RM, do Nascimento CM, Oyama LM, *et al*: **Interdisciplinary therapy improves biomarkers profile and lung function in asthmatic obese adolescents.** *Pediatr Pulmonol* 2012, **47**(Suppl 1):8–17.
68. Hedeker D, Gibbons RD, Waternaux C: **Sample size estimation for longitudinal designs with attrition.** *J Educ Behav Stat* 1999, **24:**70–93.
69. Ford AL, Hunt LP, Cooper A, Shield JP: **What reduction in BMI SDS is required in obese adolescents to improve body composition and cardiometabolic health?** *Arch Dis Child* 2010, **95**(Suppl 4):256–261.
70. Rice J, Thombs D, Leach R, Rehm R: **Successes and barriers for a youth weight-management program.** *Clin Pediatr* 2008, **47**(Suppl 2):143–147.
71. Abma TA: **Responsive evaluation in health promotion: its value for ambiguous contexts.** *Health Promot Int* 2005, **20**(Suppl 4):391–397.
72. Anto JM: **Recent advances in the epidemiologic investigation of risk factors for asthma: a review of the 2011 literature.** *Curr Allergy Asthma Rep* 2012, **12**(Suppl 3):192–200.

doi:10.1186/1471-2458-13-494
Cite this article as: Willeboordse *et al.*: Multifactorial intervention for children with asthma and overweight (Mikado): study design of a randomised controlled trial. *BMC Public Health* 2013 **13**:494.

Submit your next manuscript to BioMed Central and take full advantage of:

- **Convenient online submission**
- **Thorough peer review**
- **No space constraints or color figure charges**
- **Immediate publication on acceptance**
- **Inclusion in PubMed, CAS, Scopus and Google Scholar**
- **Research which is freely available for redistribution**

Submit your manuscript at
www.biomedcentral.com/submit

BioMed Central
