# Supplementary material for: A Multifactorial Weight Reduction Programme for Children with Overweight and Asthma: A Randomized Controlled Trial
Source: PLoS One. 2016 Jun 13;11(6):e0157158. doi: 10.1371/journal.pone.0157158 (PMC4905647; doi:10.1371/journal.pone.0157158)
Supplement: S2 File — (DOCX) [file pone.0157158.s003.docx]

**S2 FILE: STATISTICAL ANALYSES**

**Statistical primary analysis: BMI-SDS and FEV_1_% predicted**

The dependent variables for the primary analyses were BMI-SDS and FEV_1_% predicted. Participants were treated as a second-level random factor, and the first-level measurements (T0, T6, T12, T18) were included by repeated measures, with a compound symmetry covariance structure for both FEV_1_% predicted and BMI-SDS. The intervention group was included as a between-subjects covariate, and measurement as a categorical within-subject covariate with T0 as a reference category. The interaction term ‘measurement by group’ was included as a covariate. As measurements took place within a period of five months, seasonal differences may have occurred which could have influenced FEV_1_% predicted [34]. Therefore, in the analysis with FEV_1_% predicted, season of the measurement, and an interaction term of season by group were also included as covariates. Both intention to treat (with all participants who completed the measurements) and per-protocol analyses (with only participants who continued the intervention) were performed. Results are shown for intention-to-treat analyses, unless indicated otherwise.

**Statistical secondary analysis: Asthma features**

The dependent variables for the secondary analyses were: FVC% predicted, FEV_1_/FVC, TLC% predicted, ERV% predicted, asthma control, asthma-related quality of life (PAQLQ score), degree of EIB, leptin, adiponectin, ICS use and SABA use. For asthma control, PAQLQ scores, and medication use, analyses were conducted without subjects who had a high risk of developing asthma. For all analyses, two levels were used: participants and measurements (T0, T6, T12, T18). Asthma control was determined by the c-ACT for the children aged 6-12 (outcome range 0-27) and ACT score for the children aged ≥12 at baseline (outcome range 5-25). Analyses for asthma control were performed with (c)-ACT scores as the continuous dependent variable, and the ACT-group (e.g. c-ACT or ACT) as a covariate to correct for differences between the two questionnaires. Participants were treated as a second-level random factor, and the first level measurements were included by repeated measures, with a compound symmetry covariance structure for all dependent variables except PAQLQ. In the analysis with PAQLQ as a dependent variable, an unstructured covariance structure was used. An intervention group was included as a between-subjects covariate. Measurement was included as a within-subject covariate. In variables with 4 measurements (e.g. FVC% predicted, FEV_1_/FVC, asthma control, PAQLQ score), measurements were included by using dummy coding with T0 as reference category. In all analyses, the interaction term between group and measurement was included.

ICS use and SABA use were analysed with binary mixed models for longitudinal analysis (logit link function), as these are categorical dependent variables. The same independent variables as in the linear mixed models analysis models were included and a compound covariance matrix was used.

**Statistical analyses of lifestyle variables**

Differences in the lifestyle variables diet score, step count, VO_2_peak% predicted and eating behaviours (DEBQ emotional, restraint and external eating score) over time were calculated with mixed models for longitudinal analyses. Participants were treated as a second-level random factor, and the first-level measurements were included by repeated measures, with a compound symmetry covariance structure for all lifestyle variables. Measurement was included as a within-subject covariate. In variables with more than two measurements (e.g. diet score, step count), measurements were included by using dummy coding with T0 as a reference category. In all analyses, the interaction term between the group and measurement was included. Per-protocol analyses were not performed for this secondary analysis as this would have caused a considerable loss of power.

**References**

1. Nathan RA, Sorkness CA, Kosinski M, Schatz M, Li JT, Marcus P, et al. Development of the asthma control test: a survey for assessing asthma control. The Journal of allergy and clinical immunology. 2004 Jan;113(1):59-65. PubMed PMID: 14713908.

2. Liu AH, Zeiger R, Sorkness C, Mahr T, Ostrom N, Burgess S, et al. Development and cross-sectional validation of the Childhood Asthma Control Test. The Journal of allergy and clinical immunology. 2007 Apr;119(4):817-25. PubMed PMID: 17353040.

3. Juniper EF, Guyatt GH, Feeny DH, Ferrie PJ, Griffith LE, Townsend M. Measuring quality of life in children with asthma. Qual Life Res. 1996 Feb;5(1):35-46. PubMed PMID: 8901365.

4. Miller MR, Hankinson J, Brusasco V, Burgos F, Casaburi R, Coates A, et al. Standardisation of spirometry. Eur Respir J. 2005 Aug;26(2):319-38. PubMed PMID: 16055882.

5. Willeboordse M, van de Kant KD, de Laat MN, van Schayck OC, Mulkens S, Dompeling E. Multifactorial intervention for children with asthma and overweight (Mikado): study design of a randomised controlled trial. BMC Public Health. 2013;13:494. PubMed PMID: 23692648. Pubmed Central PMCID: 3682864.

6. Bongers B, Hulzebos, HJ., van Brussel, M., Takken, T. Pediatric norms for cardiopulmonary exercise testing: in relation to gender and age. 's Hertogenbosch: BOXpress; 2012.

7. Gezondheidsraad. Richtlijnen goede voeding 2006. In: Gezondheidsraad, editor: Den Haag; 2006.
