# Supplementary material for: A Multifactorial Weight Reduction Programme for Children with Overweight and Asthma: A Randomized Controlled Trial
Source: PLoS One. 2016 Jun 13;11(6):e0157158. doi: 10.1371/journal.pone.0157158 (PMC4905647; doi:10.1371/journal.pone.0157158)
Supplement: S4 File — (DOCX) [file pone.0157158.s005.docx]

**S4 FILE: POST HOC ANALYSES**

**Methods of post-hoc analysis**

The dependent variables for the post-hoc analysis were FEV_1_% predicted, FVC% predicted and (c)-ACT score. A multilevel linear mixed model with two levels (participants and measurements) was conducted. Participants were treated as a second-level random factor, and the first-level measurements were included by repeated measures, with a compound symmetry covariance structure. Measurement (T0 as reference category), BMI-SDS, leptin concentration (as a marker for the inflammatory pathway) and ERV% predicted (as a marker for the mechanical pathway) were included as within-subject covariates. The interaction terms BMI-SDS*measurement, ERV*measurement and leptin*measurement were included in the model to determine if changes in BMI-SDS, ERV and leptin were related to changes in FEV_1_% predicted, FVC% predicted and asthma control.

For the model with asthma control, ACT-group (e.g. c-ACT or ACT) was also included as a covariate. The inclusion of this covariate enabled us to correct for differences between the c-ACT questionnaire (aged 6-12 (outcome range 0-27)) and ACT score (children aged ≥12 at baseline (outcome range 5-25)).

**Results of post-hoc analysis**

None of the changes over time in BMI-SDS, ERV and leptin were related to FEV_1_% predicted, FVC% predicted or asthma control (P<0.05).

When examining data cross-sectionally, it was observed that BMI-SDS was significantly related to FEV_1_% predicted, FVC% predicted and asthma control. Participants with higher BMI-SDS scores had slightly worse lung function (FEV_1_% predicted: Estimates -6.62, (p<0.05)) and FVC% predicted: Estimates -7.74 (p<0.001)) and a lower ACT score (Estimates -0.30, (p<0.05)). A higher ERV was associated with a higher FEV_1_% predicted (Estimates 0.15 (p<0.01), and a higher FVC% predicted (Estimates 0.18, (p<0.001)), but was not related to the ACT score (p≥0.05). Leptin was not related to FEV_1_% predicted, FVC% predicted or asthma control.
