# Supplementary material for: A Multifactorial Weight Reduction Programme for Children with Overweight and Asthma: A Randomized Controlled Trial
Source: PLoS One. 2016 Jun 13;11(6):e0157158. doi: 10.1371/journal.pone.0157158 (PMC4905647; doi:10.1371/journal.pone.0157158)
Supplement: S1 Table — (DOCX) [file pone.0157158.s006.docx]

**S1 TABLE: ADDITIONAL BASELINE CHARACTERISTICS**

|  | Total group (n=87) | Intervention group (n=43) | Control group (n=44) |
| --- | --- | --- | --- |
| *Questionnaires* |  |  |  |
| Suspicion of Sleep Related Breathing Disorders according to PSQ, n/N* (%) | 37/87 (43) | 20/43 (47) | 17/44 (39) |
| Suspicion of Gastro Oesophageal Reflux disease according to GERD, n/N* (%) | 11/87 (13) | 3/43 (7) | 8/44 (19) |
| Score for strengths and difficulties questionnaire (t=12), median (IQR) | 9.0 (7.0) | 9.0 (7.0) | 9.5 (7.0) |
| Dutch eating behaviour questionnaire  External eating, median (IQR)  Emotional eating, median (IQR)  Restraint eating, median (IQR) | 8.0 (7.0)  9.0 (15.0)  10.0 (11.0) | 7.0 (6.3)  9.0 (14.3)  10.0 (9.0) | 8.0 (7.5)  9.0 (16.0)  11.0 (10.5) |
| VAS score self-rated health EQ-5D-Youth, median (IQR) | 80 (23) | 80 (26) | 80 (25) |
| Experienced wheezing episodes in the previous 12 months†, n/N* (%) | 50/68 (74) | 22/32 (69) | 28/36 (78) |
|  |  |  |  |
| *Lifestyle parameters* |  |  |  |
| Daily step count, median (IQR) | 8267 (3009) | 8358 (3273) | 8246 (3137) |
| VO_2peak_ in ml/min, mean (SD) | 2021 (583) | 2093 (630) | 1940 (522) |
| VO_2peak_ in ml/min/kg, mean (SD) | 31.2 (6.1) | 31.8 (6.0) | 30.6 (6.3) |
| Wmax, median (IQR) | 150 (62) | 152 (70) | 144 (60) |
| W%pred, mean (SD) | 108 (20) | 108 (21) | 108 (21) |
| VO2peak%pred, mean (SD) | 70.9 (12.6) | 72.1 (12.2) | 69.5 (13.1) |
| EIB†‡, n/N* (%) | 12/64 (19) | 4/31 (13) | 8/33 (24) |
| Diet score, mean (SD) | 9.6 (2.1) | 10.0 (2.1) | 9.2 (2.1) |

* n/N: Number of participants with a positive outcome for this parameter / Number of participants measured. Numbers (N) may not add up to 87, 43 and 44 respectively due to missing values or, in case of asthma related parameters (†), because those parameters are only demonstrated for asthmatic participants (not for participants at high risk of developing asthma).

‡ EIB was defined as a fall in FEV_1_%predicted of ≥10% compared to baseline after a maximal exercise test.

Abbreviations: EQ-5D-Youth: euroqol 5 dimensions youth questionnaire for quality of life, EIB: exercise induced bronchoconstriction, GERD: gastro oesophageal reflux disease, IQR: inter quartile range, PSQ: paediatric sleep questionnaire, SD: standard deviation, VO_2_peak: peak oxygen uptake during a maximal exercise test, Wmax: Maximum resistance (watt) during a maximal exercise test.
